# Fine organization of genomic regions tagged to the 5S rDNA locus of the bread wheat 5B chromosome

**DOI:** 10.1186/s12870-017-1120-5

**Published:** 2017-11-14

**Authors:** Ekaterina M. Sergeeva, Andrey B. Shcherban, Irina G. Adonina, Michail A. Nesterov, Alexey V. Beletsky, Andrey L. Rakitin, Andrey V. Mardanov, Nikolai V. Ravin, Elena A. Salina

**Affiliations:** 1 The Federal Research Center “Institute of Cytology and Genetics SB RAS”, Novosibirsk, Russia; 2 The Federal Research Center “Fundamentals of Biotechnology RAS”, Moscow, Russia; 3 Faculty of Biology, Moscow State University, Moscow, Russia

**Keywords:** 5S rDNA, BAC-clones, Polyploid wheat, 5BS chromosome, FISH

## Abstract

**Background:**

The multigene family encoding the 5S rRNA, one of the most important structurally-functional part of the large ribosomal subunit, is an obligate component of all eukaryotic genomes. 5S rDNA has long been a favored target for cytological and phylogenetic studies due to the inherent peculiarities of its structural organization, such as the tandem arrays of repetitive units and their high interspecific divergence. The complex polyploid nature of the genome of bread wheat, *Triticum aestivum*, and the technically difficult task of sequencing clusters of tandem repeats mean that the detailed organization of extended genomic regions containing 5S rRNA genes remains unclear. This is despite the recent progress made in wheat genomic sequencing. Using pyrosequencing of BAC clones, in this work we studied the organization of two distinct 5S rDNA-tagged regions of the 5BS chromosome of bread wheat.

**Results:**

Three BAC-clones containing 5S rDNA were identified in the 5BS chromosome-specific BAC-library of *Triticum aestivum*. Using the results of pyrosequencing and assembling, we obtained six 5S rDNA- containing contigs with a total length of 140,417 bp, and two sets (pools) of individual 5S rDNA sequences belonging to separate, but closely located genomic regions on the 5BS chromosome. Both regions are characterized by the presence of approximately 70–80 copies of 5S rDNA, however, they are completely different in their structural organization. The first region contained highly diverged short-type 5S rDNA units that were disrupted by multiple insertions of transposable elements. The second region contained the more conserved long-type 5S rDNA, organized as a single tandem array. FISH using probes specific to both 5S rDNA unit types showed differences in the distribution and intensity of signals on the chromosomes of polyploid wheat species and their diploid progenitors.

**Conclusion:**

A detailed structural organization of two closely located 5S rDNA-tagged genomic regions on the 5BS chromosome of bread wheat has been established. These two regions differ in the organization of both 5S rDNA and the neighboring sequences comprised of transposable elements, implying different modes of evolution for these regions.

**Electronic supplementary material:**

The online version of this article (doi: 10.1186/s12870-017-1120-5) contains supplementary material, which is available to authorized users.

## Background

The multigene family encoding the 5S rRNA genes in eukaryotes is generally organized in tandem arrays located on one or several chromosomes, separate from the genes encoding the 45S rRNA [1–3]. The repeating unit of 5S rDNA contains a 120 bp coding region and a non-transcribed spacer sequence (NTS). The former is highly conserved in structure, whereas the NTS is polymorphic in both length and nucleotide sequence. In the hexaploid genome of bread wheat *Triticum aestivum* L. (2n = 6× = 42, BBAADD, the potential donors of A, D and B genomes are *T. urartu*, *Ae. tauschii* and *Ae. speltoides* respectively) the two major unit classes are discriminated according to the NTS structure: the long unit of about 500 bp long contains a 380 bp NTS and the short unit of 400 bp has a smaller NTS of 280 bp [2, 4]. The short units of 5S rDNA have preferential localization on the chromosomes of homoeologous group 1 (arms 1AS, 1BS, and 1DS), while the long units are located on group 5 (arms 5AS, 5BS, and 5DS) [2, 5–7]. Based on the interspecific variation of the 5S rDNA NTS sequences, a number of different unit types were discriminated and assigned to conventional haplomes (genomic types) in the Triticeae [8–11].

The organization of 5S rDNA into repetitive clusters and their NTS variation have made them a popular tool for cytological and phylogenetic studies. For wheat and its relatives, a number of studies have been carried out: phylogenetic analysis using individually cloned copies of 5S rDNA [10, 12–14], as well as multiple cytological studies of 5S rDNA chromosomal localization [11, 15–19]. Of great interest is the study of rRNA gene organization in allopolyploid Triticeae in comparison to their diploid ancestors. Using synthetic allopolyploid *Triticum* x *Aegilops*, it was found that reorganization of 5S rDNA occurred soon after the formation of allopolyploids [20, 21]. This reorganization was reproducible in both synthetic allopolyploids and natural wheat allopolyploids with a similar genomic constitution.

Despite the progress in total genome sequencing and appearance of the first version of the reference sequence for the bread wheat variety Chinese Spring (IWGSC RefSeq v1.0) (http://www.wheatgenome.org/), some long arrays of tandem repeats, including the 5S rDNA, still require additional analysis [22].

In this work, we described the detailed structure of two 5S rDNA-containing genomic regions belonging to chromosome 5BS. 454/GS FLX platform, BAC-End- and IonTorrent sequencing were exploited to study the organization of 5S rDNA arrays and adjacent regions. In combination with fluorescent in situ hybridization (FISH), this analysis allowed us to establish the chromosomal locality of different units of 5S rDNA and their surrounding “landscape”. FISH of 5S rDNA NTS sequences on metaphase chromosomes of different wheat polyploids and their diploid progenitors was applied to study the changes in chromosomal organization of the studied 5S rDNA loci throughout the course of their evolution from diploid precursors to hexaploid wheat.

## Methods

### Plant material

We used *Triticum aestivum* (2n = 6× = 42, AABBDD), var. Chinese Spring; *T. timopheevii* (2n = 4× = 28, AAGG)*,* K-38555; *Ae. speltoides* (2n = 2× = SS), K-389; *Ae. tauschii* (2n = 2× = DD), К-1662; and *T. urartu* (2n = 2× = AA), IG45298*. The accessions were from the wheat germplasm collection of the N.I. Vavilov All-Russian Institute of Plant Genetic Resources RAN (St Petersburg, Russia) and genbank ICARDA (Syria), and were maintained at the Institute of Cytology and Genetics.

### BAC-library screening and DNA isolation from individual BAC-clones

The 5BS-specific BAC-library of *T. aestivum* (43,776 BAC-clones, mean insert size 122 Kbp, 15-times coverage of 280 Mbp length chromosome arm) was obtained from the Institute of Experimental Botany (Olomouc, Czech Republic), kindly provided by Professor J. Doležel. A copy of the library is maintained at the Institute of Cytology and Genetics SB RAS at -80 °C. For BAC-library screening we ran PCR analysis of 2D BAC-pools with primers specific to 5S rDNA coding sequences (5SrDNA_F: 5′-GAGAGTAGTACTAGGATGGG-3′; 5SrDNA_R: 5′-GGAGTTCTGACGGGATCCGG-3′). PCR was performed in a 20 μl reaction mixture containing 0.5 μl of cell culture as a template, 0.25 pM of specific forward and reverse primers, 2 μl of PCR buffer (65 mM Tris-HCl, pH 8.9; 1.5 mM MgCl_2_; 16 mM (NH_4_)_2_SO_4_; 0.05% Tween 20), 0.2 mM of each dNTP and 1 unit of *Taq* DNA polymerase. After initial denaturation at 94 °C for 4 min, 35 cycles were run at 94 °C for 30 s, 55 °C for 30 s, and 72 °C for 30 s. PCR fragments were separated by electrophoresis on 1% agarose gels.

DNA of selected BAC clones was isolated using the NucleoSpin 96 Flash kit (Macherey-Nagel, Germany).

### Sanger BAC-end and IonTorrent sequencing

The BAC-end sequences for selected BAC-clones were obtained with the universal M13 Reverse (5′-CAGGAAACAGCTATGAC -3′) and T7 forward (5′-TAATACGACTCACTATAGGG-3′) primers using the BigDye3.1 Terminator kit (Applied Biosystems, USA). Each 20 μl reaction contained ~200 μg of BAC-DNA, 1.5 μl of BigDye 3.1, 0.25 pM of specific forward, or reverse primer, 4 μl of5X buffer and deionized water. After preliminary denaturation at 95 °C for 5 min, 80 cycles were run at 95 °C for 30s, 55 °C for 15 s, and 60 °C for 4 min. The reaction products were precipitated using ethanol, and separated in a 3730XL DNA Analyzer (Perkin Elmer Cetus, USA).

BAC clones marked by 5S rDNA were included in the pool of 134 BAC-clones belonging to different locations of chromosome 5BS, and were collectively sequenced as one sample on an IonTorrent platform (Thermo Fisher Scientific). The sequencing and assembly were described in Nesterov et al. [23].

### Pyrosequencing and assembly of BAC clones

The selected BAC-clones of the 5BS chromosome were sequenced into two pools. BAC pool 52 consisted of 6 clones including 5S rDNA-tagged clones TaaCsp5BS010O13 and TaaCsp5BS025F09. Ten overlapping clones of the 5BS chromosome (http://www.wheatgenome.org/), including BAC TaaCsp5BS096G09 with 5S rDNA sequences, were 89 pool. BAC pools were shotgun sequenced using a GS FLX/454 pyrosequencing platform (Roche). Sequence data of clone TaaCsp5BS096G09, as a part of ctg4 have been made available (https://urgi.versailles.inra.fr/gb2/gbrowse/wheat_phys_5BS_v1/, https://wheat-urgi.versailles.inra.fr/Seq-Repository/Assemblies).

For both pools, a library of random BAC-fragments with sizes ranging from 400 to 1000 bp was created. Also, for both pools a paired-end library of 6–10 kbp was constructed. The DNA sequencing was conducted according to Sequencing Method Manual GS FLX+ series with Titanium L+ Kit (Roche). The reads, belonging to *E.coli* DH10B and pIndigoBAC-5 vector were removed. The selected shotgun and paired-end reads were de novo assembled into contigs (for shotgun reads) and scaffolds (for paired-end reads) using the GS DeNovo Assembler V 2.9.

### Identification and subsequent analysis of 5S rDNA-tagged sequences in selected BAC-clones

First, we searched for 454-contigs longer than 700 bp (shotgun reads) and scaffolds (paired-end reads) tagged by 5S rDNA, using: (1) BLASTn searches with sequences of complete 5S rDNA units (both coding and non-transcribed sequences); and (2) BLASTn searches with obtained BES (BAC-end sequences) [24]. Additionally, for verification and elongation of some contigs we used the IonTorrent BAC-clone sequencing data and PCR product sequences obtained with specific primers (Additional file 1: Table S1). The 6 selected 5S rDNA-containing contigs and scaffolds (hereafter named “5SrDNA_fragments”) were annotated by BLASTn search in TREP (http://botserv2.uzh.ch/kelldata/trep-db/index.html) and NCBI non-redundant nucleotide databases (https://blast.ncbi.nlm.nih.gov/Blast.cgi). The nucleotide sequences of 5S rDNA fragments were deposited in GenBank under the accession numbers: MF467437 for pool_52 (5 unordered pieces) and MF467438 for pool_89.

Primers were developed with the Primer3 program [25]. To test for the degree of BAC-clone overlap, 3 Insertion Size Based Polymorphism (ISBP) primer pairs were designed and tested on the individual BAC-DNA templates (Additional file 2: Figure S1). ISBP exploits knowledge of the sequence flanking a TE to PCR-amplify a fragment spanning the junction between the TE and the flanking sequence [26].

To date the LTR-retrotransposon insertion events for the autonomous transposable elements, we analyzed the nucleotide divergence rate between two LTRs in cases when both LTRs were present in the element’s structure. To determine the LTR boundaries, each element was compared with itself using Blast2seq (https://blast.ncbi.nlm.nih.gov/Blast.cgi). In addition, the presence of the characteristic motifs, 5′-TG-3′ and 5′-CA-3′, at the beginning and end of each LTR, respectively, was taken into account. Each pair of LTRs was aligned using the ClustalW algorithm within the MEGA4 program [27]. Sequence divergence was calculated using the Kimura two-parameter method [28] with complete deletion option. To convert this term into the insertion date, we used the following equation: *T* = *D*/2*r*, where *T* is the time elapsed since the insertion; *D*, the estimated LTR divergence; and *r*, the substitution rate per site per year [29]. We applied a substitution rate of 1.3 × 10^−8^ mutations per site per year for the plant LTR retrotransposons [30].

### 5S rDNA sequence alignment and cluster analysis

A search of 5S rDNA sequences was performed in contigs and scaffolds and also in primary reads employing BLASTn search with 5S rDNA coding sequence as a query. In order to find spacer sequences between 5S rDNA coding regions we identified all reads containing two or more copies of 5S rDNA and extracted the sequences located between 5S copies. The coding and spacer sequences were analyzed separately. The alignment of 5S rDNA spacer sequences was done by MUSCLE program [31] included into MEGA 4 software. The calculation of 5S rDNA coding sequence divergence was done using the pairwise-deletion option and Kimura-2-parameter model. The cluster analysis of 5S rDNA coding sequences and spacers was performed with the CD-HIT program [32]. A neighbor-joining phylogenetic tree was obtained with the MEGA4 program using the pairwise deletion option with bootstrap replicates of 500.

### Secondary 5S structure analysis

Secondary structure modelling was carried out using an online tool at the RNAfold web server (http://rna.tbi.univie.ac.at/). The secondary structures were based on minimum free energy (MFE) calculations using a loop-based energy model and the dynamic programming algorithm introduced by Zuker and Stiegler [33]. For both groups of 5S rDNA (pool_52 and pool_89 representing short and long units, respectively) a consensus model of secondary structures was constructed. The program setting was as follows: isolated nucleotides were avoided; vote for dangling energies on both sides of a helix in any case.

### Fluorescence in situ hybridization (FISH)

Metaphase chromosome preparation, FISH and chromosome identification were performed according to Salina et al. [34] with minor modifications. The total number of analyzed metaphases from individual plants for each probe was 15–30. For FISH, we used the PCR-amplified sequences Short5S and Long5S (94 bp and 131 bp, respectively). The probes were amplified from BAC-clones TaaCsp5BS010O13 and TaaCsp5BS096G09 by PCR with specific primers Short5S-F (5′-GCGTGCACTGGTGCGGTTGAG-3′) and Short5S–R (5′- GACGATTGCACATTGCTTTGGC-3′); Long5S-F (5′-GGAAAAAACTCGTGTTGCTGC -3′) and Long5S–R (5′-CTCACTACCATTACAACCGTTC -3′) using the following program: 35 cycles at 94 °C for 45 s, at 55 °C for 45 s, at 72 °C for 30 s. The primers were designed to the representative spacer sequences of Short5S and Long5S types (determined by cluster analysis with a threshold of 98% identity), and obtained single band PCR product for each type was verified by sequencing. The mean overall sequence divergence calculated for presumable amplification regions of Short5S and Long5S 5S rDNA sequences (obtained from 454 pyrosequencing) corresponded to 0.8–0.9% for both probes. The divergence level between Short5S and Long5S probes at their complete length was 45%, wherein the small region of 62 bp has 84% identity between probes and there was no identity for the remaining sequence.

The PCR-derived probes were labeled with biotin or digoxigenin. Biotinylated probes were detected with fluorescein avidin D (Vector Laboratories, United States). The hybridization signal was enhanced using fluorescein anti-avidin (Vector Laboratories, United States). The digoxigenin-labeled probes were detected with antibodies to anti-digoxigenin-rhodamine (Fab fragments, Sigma-Aldrich, United States). The preparations were embedded in Vectashield mounting medium (Vector Laboratories), containing 0.5 μg/ml DAPI (4′, 6-diamidino-2-phenylindole, Sigma-Aldrich, United States) for chromosome staining. The chromosomes were examined with an Axioskop 2 Plus (Zeiss) microscope and recorded with a VC-44 (PCO) CCD camera.

To identify chromosomes carrying signals, we used the probes pSc119.2 [35] and pAs1 [36].

The work was performed at the Collective Center for Microscopic Analysis of Biological Objects (SB RAS, Novosibirsk).

## Results

### Identification, sequencing and assembly of 5S rDNA tagged BAC-clones

The three 5S rDNA-containing BAC-clones TaaCsp5BS010O13, TaaCsp5BS025F09 and TaaCsp5BS096G09 were isolated from the 5BS-specific BAC-library of *T. aestivum* var. Chinese Spring. Pyrosequencing of these BAC-clones was performed and assembled as described in the Methods. A summary for the sequencing and assembling data is presented in Table 1.

**Table 1:** The dataset obtained from shotgun pyrosequencing of pool 52 and pool 89

	Pool_52: 6 BAC-clones	Pool_89: 10 BAC-clones
5S rDNA-tagged clones	TaaCsp5BS010O13, TaaCsp5BS025F09	TaaCsp5BS096G09
Number of bases, bp	23,529,114	22,416,976
Average contig coverage	42	24
Estimated 5S rDNA coding sequence coverage	3600	1500
Number of contigs >500 bp	43	116
Total length of contigs, bp	433,354	650,514
Average contig size, bp	10,078	5607
N50 contig size, bp	52,492	9364
Largest contig, bp	10,664	56,645

The 5S rRNA gene number in BAC pools was approximately calculated. For pool 52 the 5S rDNA sequence coverage was established as 3600, whereas the contig coverage by 454-reads was 42, thereby the 5S rDNA copy number was assessed as 86. The 5S rDNA sequence coverage for pool 89 was 1500, and the contig coverage 24, which gives the 5S rDNA copy number as 63.

In order to increase the assembly quality for pool 52, paired end 454-sequencing was performed (Additional file 3: Table S2). Consequently, the assembled reads were arranged into 11 scaffolds with lengths ranging from 2159 to 164,054 bp. It is noteworthy that the lengths of the individual scaffolds are comparable with the BAC-clone length.

The data of shotgun and paired-end pyrosequencing were used for analysis of 5S rDNA-tagged genomic fragments from 5BS chromosome of the bread wheat (Additional file 1: Table S1).

### Structural organization of 5S rDNA-tagged sequences of chromosome 5BS

For identification of 5S rDNA-tagged long genomic sequences in pool 52 and pool 89 contigs, we performed a BLASTn search using contigs longer than 700 bp as query. As a result, we obtained six 5S rDNA-containing contigs (hereafter named “5S rDNA fragments”) with lengths from 2503 to 52,840 bp (Table 2; Additional file 1: Table S1).

**Table 2:** Composition of identified 5S rDNA-tagged genomic fragments in pools of BAC-clones 52 and 89. The TE element descriptions are given according to the Triticeae repeat sequence database (TREP) classification [64]

	Length, bp	TE composition (length, bp)	5S rDNA
Pool_52			
5S rDNA Fragment 1	10,801	RLG_Laura, 5895 bp	1 hit, 227 bp
5S rDNA Fragment 2	52,840	RLG_Danae, 15,807 bp RLG_Fatima, 9148 bp DTC_Jorge, 10,482 bp RLG_WHAM, 4974 bp Unnamed_DTC, 1589 bp RLX_Ginger, 4510 bp DTM_Deimos, 399 bp RLG_Egug, 602 bp DTM_Sherlock, 1917 bp RLG_Laura, 1000 bp	1 hit, 363 bp
5S rDNA Fragment 3	39,574	RLG_Fatima, 8699 bp RLG_Laura, 11,838 bp RLX_Xalax, 13,537 bp	4 hits, 1924 bp
5S rDNA Fragment 4	30,284	RLG_Wilma, 7007 bp RLG_Sabrina, 6998 bp RIX, unnamed, 1251 bp RLG_Fatima, 2084 bp RLG_Daniela, 9838 bp	1 hit, 1429 bp
5S rDNA Fragment 5	4415	RLG_Fatima, 2771 bp DTC_Jorge, 1477 bp	1 hits, 162 bp
Pool_89			
5S rDNA Fragment 6	2503	RLG_Nusif, 2411 bp	1 hit, 92 bp

**Fig. 1:**
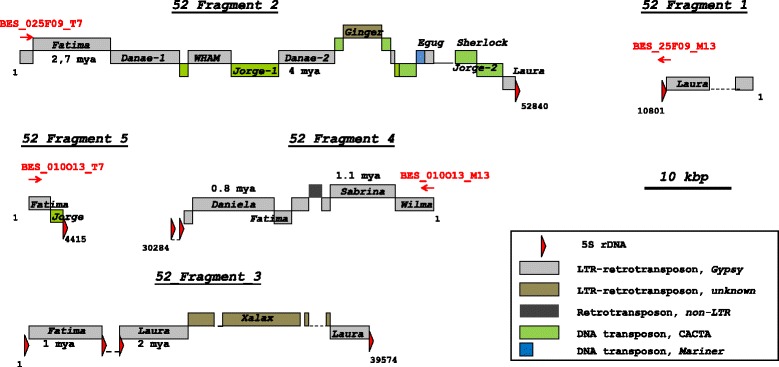
Structural organization of 5S rDNA-containing 5S rDNA fragments belonging to one genomic region of 5BS chromosome of *T. aestivum.* These 5S rDNA fragments originated from BAC-clones TaaCsp5BS010O13 and TaaCsp5BS025F09. The positions of BAC End Sequences are indicated by red arrows with the above designation of affiliation to individual BAC-clone. The date of LTR-retrotransposons insertions (mya – million years ago) is indicated under the box presenting the corresponding TE

For pool 52, five 5S rDNA fragments were identified and just one 5S rDNA fragment was found for pool 89. The 5S rDNA fragments were annotated using BLASTn searches against the Triticeae transposable element database (TREP) and NCBI nucleotide database (Table 2). All 5S rDNA fragments consist of a combination of transposable elements (TE) and 5S rDNA. No unique sequences representing potential genes or pseudogenes were found. The TEs in 5S rDNA fragments appear as single elements (or their component parts) interspersed within the 5S rDNA, or as multiple, nested TE insertions (predominantly LTR-retrotransposons). The 5S rDNA sequences, mainly located at the ends of 5S rDNA fragments, represent partial sequences from 92 bp up to complete units. In 5S rDNA fragment 3, the 5S rDNA was introduced within the fragment sequence (Fig. 1). In just one case (5S rDNA Fragment 4) the LTR-retrotransposon *Fatima* was inserted into the coding sequence of 5S rDNA, while in all other cases the TEs were inserted into spacer sequences.

The overlap between BAC-clones TaaCsp5BS010O13 and TaaCsp5BS025F09 from pool 52 was tested by PCR, with specific primers designed to TE insertions (ISBP-markers) (Additional file 2: Figure S1). We showed that BAC clone TaaCsp5BS025F09 completely overlaps with TaaCsp5BS010O13, which is apparently longer. Since pools 52 and 89 consist of different groups of overlapping BAC clones, we can conclude that pool 52 and pool 89 are attributed to different genomic locations on chromosome 5BS of *T. aestivum*.

The analysis of pool 52 and pool 89 sequences implies their different structural organization. In pool 52, 5S rDNA sequences were interrupted by multiple TE insertions, whereas in pool 89 only one TE insertion was observed on one of the flanking regions (Table 2). The repetitive 5S rDNA units of pool 89 were apparently merged in the assembly process, suggesting a single tandem cluster of 5S rRNA genes adjoining the Nusif LTR-retrotranposon. This is confirmed by the correspondence of pool 89 to the 5BS Illumina pseudomolecule (IWGSC RefSeq v1.0, http://www.wheatgenome.org/). Simultaneously, no correspondence between BAC-clones TaaCsp5BS010O13 and TaaCsp5BS025F09 from pool 52 and the Illumina data was found.

### 5S rDNA coding and spacer sequence analysis

The 5S rDNA coding and spacer sequences were extracted from the shotgun 454-reads of pool 52 and pool 89. The 5S rDNA sequences were assembled into two files for NTS, and the coding sequences for each pool analyzed separately.

A total of 1511 and 615 copies of 5S rDNA sequence were identified in reads obtained for BAC pools 52 and 89, respectively. For analysis, we removed 5S rDNA sequences which occurred less than 5 times since these are likely to be a result of sequencing errors. Cluster analysis with 99% sequence identity threshold yielded 30 types of complete 5S rDNA coding sequences for pool_52 and 21 type for pool_89. The sequences were aligned (Additional file 4) and mean overall sequence divergence was calculated: for pool 52, the value was 2.8% and for pool 89–1.1%. Thus, the coding sequences in pool 52 showed more sequence heterogeneity than in pool 89.

**Fig. 2:**
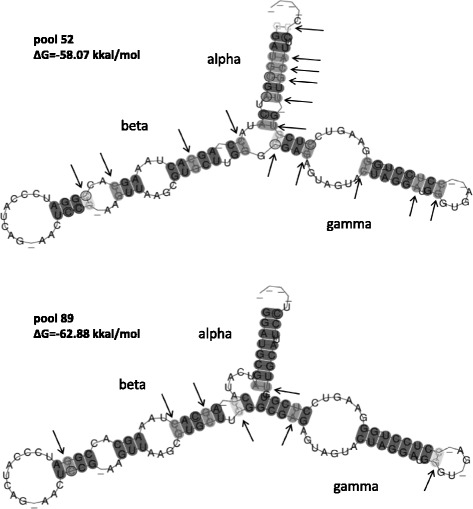
Presumed 5S rRNA secondary structures. Arrows indicate that a base-pair cannot be formed in some sequences of the alignment. Domains of 5S rRNA follow the nomenclature of [65]

To evaluate the functionality of the rRNA genes at the different loci of the 5BS chromosome, we used a special program that predicted a consensus secondary structure for each pool of RNA sequences and gave an accompanying estimate of its thermodynamic stability (see Methods). In fact, both pools produced identical molecular shapes, consisting of three functional domains (Fig. 2). The thermodynamic stability of the 5S RNA for pool 89 was slightly higher than for pool 52, although in both cases it is quite high in comparison with what is typical for functional genes (~ 50 kcal / mol) [37]. Nevertheless, both pools differed significantly in the number of incompatible pairs (15 for pool 52 and 7 for pool 89). In other words, there are approximately twice as many gene sequences in pool 52 producing RNA structures with unpaired nucleotides after folding.

Sequences of pool 52 and pool 89 differ in the structure of NTS types. In pool 89 we found 27 complete NTS sequences. The complete sequences, with mean length of 368 bp and low sequence heterogeneity, as well as partial spacer sequences, were subjected to cluster analysis (Additional file 5). Three clusters were obtained at 98% sequenced identity threshold. For the alignment we took representative sequences of these three clusters (Additional file 6). All pool 89 NTS sequences were attributed to LongS1 type (as designated by Baum and Bailey [10]).

In pool 52 we found 144 complete NTS sequences which were divided into two major clusters of 88 and 10 sequences (under a threshold of 98% identity) with a mean spacer length of 295 bp, and 39 minor clusters containing from one to four sequences of 207 to 390 bp (Additional file 5). The 22 NTS originating from minor clusters had large insertions from 30 to 95 bp, that cannot be sequencing errors.

**Fig. 3:**
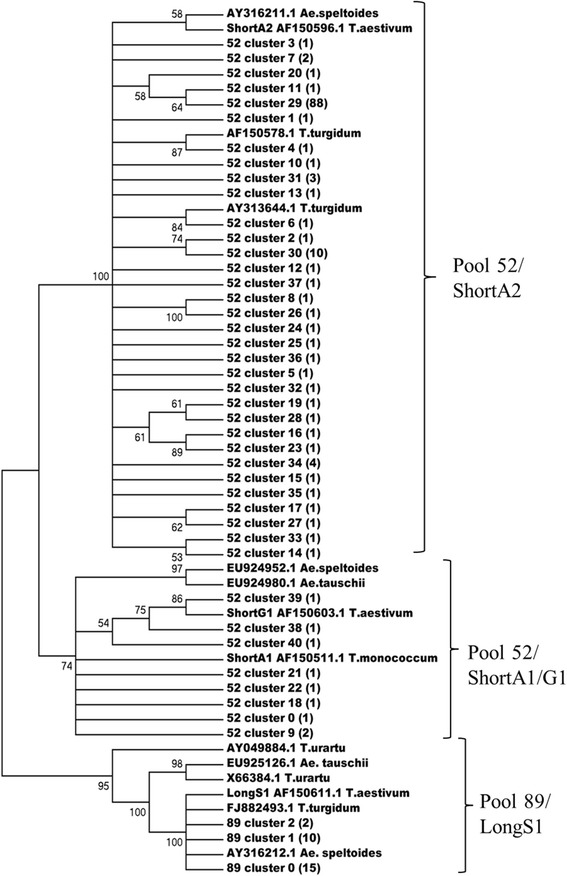
Neighbor-joining phylogenetic tree of representative clusters of 5S rDNA, sequenced from pool 52 and pool 89. The phylogenetic tree was constructed using a CLUSTALW multiple alignment for the 5S rDNA spacer nucleotide sequences (neighbor-joining method, pairwise deletion option, 500 bootstrap replicates). Bootstrap support over 50% is shown for the corresponding branches. The number of spacer sequences united in the cluster is indicated in brackets. The ShortA1, ShortG1, ShortA2 and LongS1 unit types used are also shown [10]. The branches are designated as ShortA1, ShortA2 and LongS1 based on the presence of the relevant 5S rDNA type

Construction of the neighbor-joining tree showed that most NTS sequences from pool 52 (133) correspond to the ShortA2 type (in agreement with the classification of Baum and Bailey [10]), whereas 8 minor NTS clusters are closer to ShortA1 and ShortG1 units. All 27 NTS sequences from pool 89 correspond to the LongS1 type (Fig. 3).

The overall mean sequence divergence with the pairwise deletion option between the sequences of tree branches is 21% between ShortA2 and ShortA1, 24% between ShortA1 and LongS1, and 29% between ShortA2 and LongS1. Within each of tree branches the mean overall divergence values were: 8% for ShortA2, 11% for ShortA1 and 12% for LongS1. The alignment of representative pool 52 cluster sequences derived from both ShortA2 and ShortA1 branches of the tree (Additional file 7) showed that the major representative cluster of 88 spacer sequences was completely identical to the ShortA2 type; while the two minor clusters from ShortA1 showed 19% divergence in comparison with ShortA1 and ShortG1.

To check the affinity of pool 52 and pool 89 NTS sequences to the presumed wheat progenitors, *T. urartu*, *Ae speltoides* and *Ae. tauschii*, we first performed the GenBank BLAST search with representative sequences 89_cluster_0 and 52_cluster_29 as queries. Among the 5S rDNA sequences of diploids, the *Ae. speltoides* sequence AY316211.1 is a single highly homologous sequence (99% of identity) to the ShortA2 type, whereas the remaining 29 *Ae. speltoides* sequences from GenBank showed only the 75–76% of identity to this type. The similar level of homology to ShortA2 type is characteristic of 5S rDNA from *T. urartu*, *T. monococcum* and *Ae. tauschii*.

The LongS1 type (pool 89) has a greater affinity to the corresponding sequences of 5S rDNA from diploids: for *Ae. speltoides* 5S rDNA there are 18 GenBank sequences with identity from 91 to 99%, for *Ae. tauschii -* 19 sequences with 82–87% and 30 sequences with 82–84% homology from *T. urartu* and *T. monococcum*. It should be noted that both LongS1 and ShortA2 show high identity to numerous 5S rDNA sequences from tetraploid *T. turgidum*. We added the best-matching GenBank 5S rDNA sequences of *T. urartu*, *T. monococcum*, *Ae. speltoides*, *Ae. tauschii* and *T. turgidum* to the phylogenetic tree (Fig. 3).

### FISH of 5S rDNA NTS probes

For FISH, we used two probes representing the major type 5S rDNA spacer sequences for pool 52 and pool 89. Previously, the complete 5S rDNA unit sequences were usually used as a FISH probe, thus Mukai and coauthors [7] used the rye 5S rDNA probe pScT7, and Badaeva et al. [15, 38] used the clone pTa794 [39]. Baum and coauthors [11] assessed whether the different *Triticeae* 5S rDNA units that were assigned to haplomes could be used as a FISH- probe for tracing the origin of polyploid wheats from their diploid progenitors, or chromosomal remodeling during speciation. But interpretation of results in these cases is rather difficult due to a high level of cross-homology between different units, especially at their coding regions and 5′- and 3′- ends of NTS. Therefore, for greatest specificity in the FISH signal, we designed specific primers allowing the amplification of a short sequence within NTS to be used as a probe. The probe Short5S is 94 bp long from a major cluster (52_cluster_29) and the probe Long5S is - 131 bp, from representative copies of 5S rDNA of pool 89 (Additional file 6, Additional file 7).

**Fig. 4:**
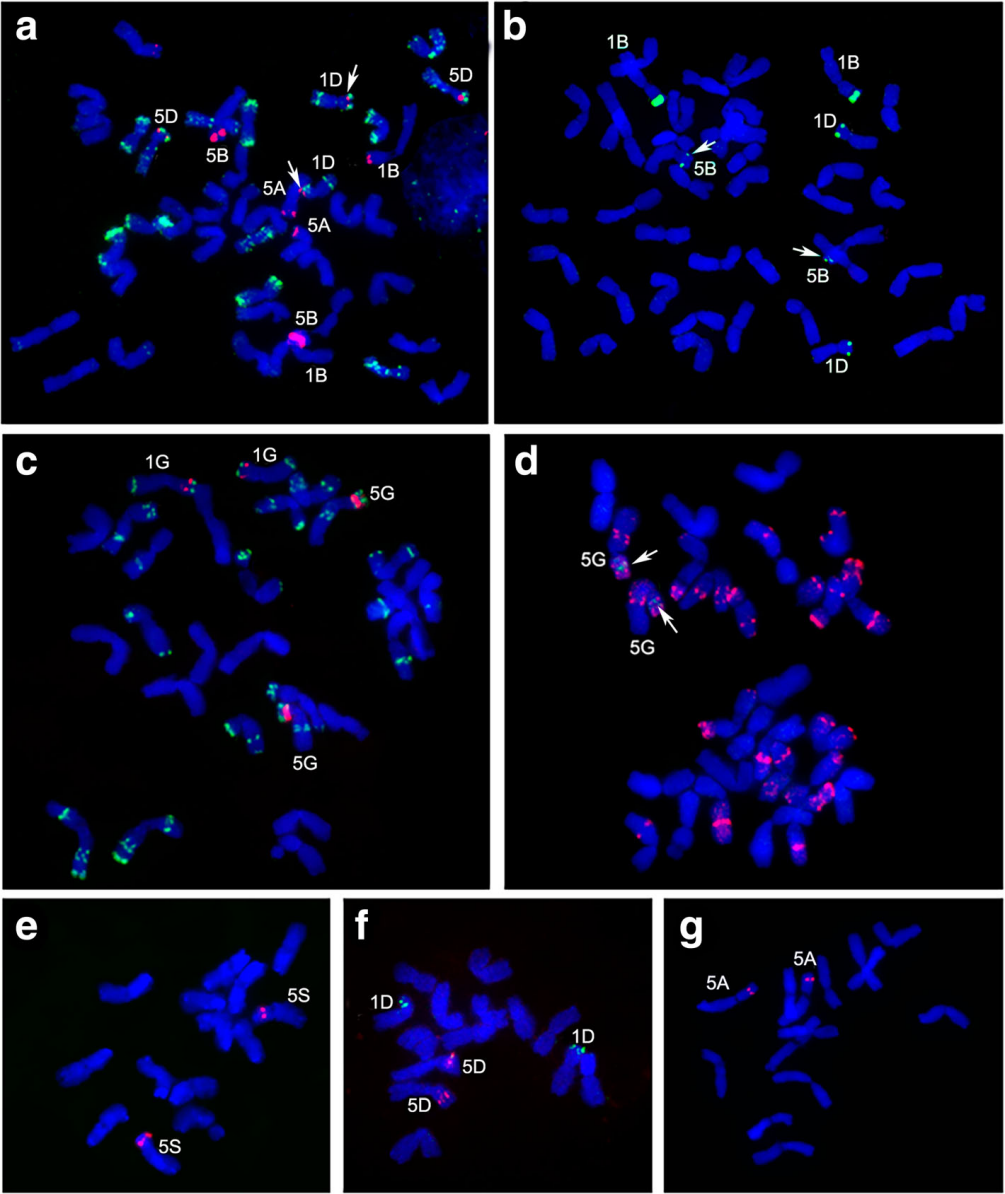
FISH to mitotic metaphase chromosomes. **a, b** *T. aestivum* cv Chinese Spring, (**c, d**) *T. timopheevii*, (**e**) *Ae. speltoides*, (**f**) *Ae. tauschii*, and (**g**) *T. urartu*. Probe combinations used were: (a) Long5S (red) and pAs1 (green), (b) Short5S (green), (c) Long5S (red) and pSc119.2 (green), d) Short5S (green) and pSc119.2 (red), (e, f, g) Long5S (red) and Short5S (green). Arrows show sites with weak signal intensity

Figure 4 represents the distribution of both probes on *T. aestivum* chromosomes. Probes Short5S and Long5S had different chromosomal locations: 10 hybridization sites on the distal parts of chromosomes 5BS, 1BS, 5DS, 5AS, 1DS were revealed for Long5S and the strongest signal was at 5BS (Table 3). Short5S showed the strongest hybridization signals on 1BS and weak signals on 1DS and 5BS. Tetraploid wheat *T. timopheevii* also had strong Long5S and weak Short5S signals on chromosome 5GS, and weak Long5S signal on chromosome 5AS. FISH of Short5S and Long5S to chromosomes of diploid species *T. urartu*, *T. monococcum*, and *Ae. speltoides* revealed no sites for Short5S hybridization*. Ae. tauschii* have both sites of 5S rDNA units, Short5S on 1DS and Long5S on 5DS. It should be noted that in polyploid wheats, Short5S units have less sites of localization than Long5S, but in most cases localization is close to Long5S on chromosomes. Copy number variation or level of 5S rDNA unit divergence may be the cause of differences in signal intensities between Short5S and Long5S in one chromosome loci. As shown in this study for chromosome 5B, the differences in signal level of Short5S and Long5S are connected with a high level of divergence of Short5S compared to Long5S (Fig. 2), despite approximately the same number of copies of these genes in the 5BS loci (84 and 75, Table 1).

**Table 3:** FISH signal distribution and comparative intensity on chromosomes of wheat and wheat relatives

Species	Long5S	Short5S
*T. aestivum* (AABBDD)	5BS > 1BS > 5DS > 5AS > 1DS	1BS > 1DS > 5BS (very weak)
*T. urartu* (A^u^A^u^)	5AS	–
*T. monococcum* (A^m^A^m^)	5AS	–
*Ae. speltoides* (SS)	5SS	–
*Ae. tauschii* (DD)	5DS	1DS
*T. timophevii* (AAGG)	5GS > 5AS	5GS – very weak

## Discussion

According to the model of “concerted evolution” reviewed in Nieto Feliner et al. [40], mutations within rRNA genes are rapidly spread across the arrays leading to intragenomic homogeneity of rDNA units. The conservation and concerted evolution of rRNA genes within separate arrays has been demonstrated in some works [41, 42]. On the other hand, the data has also accumulated showing intra- and interspecific variation in location and number of rDNA sites [43–46]. However, due to technical difficulties related to sequencing long clusters of tandem repeats [22], there is little information concerning the structural organization of separate rDNA loci. Obtaining such information is much more difficult in plants than animals due to the polyploid nature of plant genomes and higher occurrence of repetitive elements.

As shown here, the 5BS chromosome of *T. aestivum* contains two regions of 5S rDNA, showing contrasting types of structural organization. The first of the regions (pool 89) appears to have a “classical” organization, forming a single cluster of tandemly-repeated long units of 5S rDNA, which on one side borders the region representing mobile elements. The second region (pool 52) consists of at least 5 clusters of short units of 5S rDNA (Fig. 1), interrupted by blocks of insertions made up of mobile elements. There are also significant differences between regions in the repeating units of the 5S rDNA itself. Each region is characterized by its own type of units, for example, pool 89 contains approximately 63 units highly similar to each other in both the coding and NTS parts and homologous to LongS1 type of units [10]. Pool 52 contains about 86 units of 5S rDNA, which are characterized by a higher degree of heterogeneity in the coding (overall mean divergence of 2.8%) and in the NTS parts. Most spacers in pool 52 are of type ShortA2, and about 8% of sequences are closer to type ShortA1.

A high saturation of the latter region with TE suggests their involvement in transposition of rDNA units from various genomic locations to this region. Supporting this idea, in separate populations of *Aegilops speltoides* Tausch. additional 5S rDNA sites appeared during meiosis in conjunction with En/Spm transposon clusters [17]. Transposon elements of the CACTA group sometimes contain sequences similar to 5S rDNA genes [47]. Another abundant class of mobile elements, LTR retrotransposons, is frequently associated with clusters of rDNA, including 5S rDNA [48, 49].

Several mechanisms may be proposed to explain the variation apparent in the loci of rDNA:

1. Variability in these loci (deletions, amplifications, translocations) could result from homeologous recombination, conversion, and unequal crossing-over [50, 51]. In this case, the mobile elements may serve as recombination hotspots. Transposon-mediated disruptions and chromosomal recombinations played an important role in the reorganization of rDNA in allotetraploid *A. suecica* [52].
2. Multiplication of 5S rRNA genes and their integration into other areas of the genome may be explained by a mechanism similar to retrotransposition. These genes use the same machinery for their transcription (RNA polymerase III, promoter) as some retroelements, like SINE, do [53]. In addition, some authors found a unique class of retroelements which use promoter adopted from the inserted 5S rDNA for their own propagation [54].
3. 5S genes may spread into new positions of the genome via extrachromosomal replication. Extrachromosomal circles of rDNA have been found in such diverged taxa as humans, *Xenopus laevis*, and some plants [55–57].

For cytological analysis of 5S rRNA genes we used specific probes Long5S and Short5S for the central parts of NTS (Fig. 4; Table 3). The location of the Long5S probe showed good correlation with data obtained earlier for the distribution of 5S rDNA units in diploid and polyploid wheat genomes, which was obtained using pScT7 and pTa794 probes containing coding parts and NTS [7, 38] and Baum et al. [11]. The Short5S probe showed a very distinct localization. The most interesting observations are the absence of a signal for Short5S on the chromosomes of *T. urartu* and *Ae. speltoides* (Fig. 4e, g), the most probable donors of A- and B- genomes, respectively [58, 59], and its presence on the 1DS chromosome of *Ae. tauschii* (Fig. 4f), the donor of D-genome. As for polyploid species, colocalization of long and short 5S rDNA units was revealed on chromosomes 1BS, 1DS, 5BS, 5GS (Fig. 4a-d). Сhromosomes 5DS and 5AS of studied polyploid wheat displayed signal only for Long5S probe, however, the presence or absence of Short5S units in these chromosomes should be verified by other methods. According to our results, the Short5S probe showed an intense signal on the chromosome 1BS of bread wheat, and a weak signal on chromosome 5BS - likely corresponding to pool 52. Such a variation in organization of 5S rDNA in diploids and corresponding polyploids allows us to suggest that the diploid wheat progenitors contain both Short and Long 5S gene families, while their abundance, diversity and chromosome location varies significantly, especially, in the case of Short 5S units. Thus, modern diploid accessions may contain different spectrum and copy number of 5S rRNA genes than the real genome donors at the time of allopolyploidization.

Variation in rDNA loci is often enhanced through polyploidy and interspecies hybridization [20, 60–62]. However, the appearance of new 5S rDNA sites or complete disappearance of parental sites from the new synthesized allopolyploids were not revealed, only quantitative changes presumably associated with reduction of 5S rDNA copy number in a separate sites [20]. These changes being established at the early stages of polyploid formation tend to persist on the following stages of evolution. This supports by the fact of similarity of rDNA patterns in synthetic and natural allopolyploids with similar genome constitution [20]. Here, we estimated the timing of insertions of mobile elements in the studied regions of chromosome 5BS and revealed that these events occurred 2.7–4.0 MYA (Million years ago). In this regard, we can assume that 5S rDNA sites in the ancestor of diploid *Triticum* species were in the 1st and 5th chromosomes and in the course of polyploid wheat evolution there was a gradual structural divergence of separate sites, including deletion of a certain part of NTS leading to the appearance of a pool of short 5S rDNA units specific for wheat polyploids. This assumption is supported by a high abundance of the ShortA2 family, which constitutes a bulk of pool 52, among polyploid wheat species.

Both pools of 5S rRNA genes studied here have identical consensus secondary RNA structures (Fig. 2) However, they display different levels of conservation. Pool 52 likely produces more nonfunctional RNA molecules due to mutations in the gene sequences. The variation in NTS sequences of pool 52 is much higher compared to pool 89. Therefore, the latter is either under stronger selection constraints or has a reduced efficiency in interlocus recombination. This raises an important question regarding the cause of such a difference in the rate of evolution of various rDNA loci, and the related issue of their differential expression and transcriptional regulation.

It is known that the majority of rRNA genes are inactive in plant and animal genomes, or only become activated in certain developmental stages [63]. Inactive copies of genes can evolve in accordance with the neutral mechanism of evolution and undergo gradual destruction as a result of recombination events, insertions of mobile elements, etc., which we observed here in the locus of strongly diverged short units. However, in order to distinguish expressing copies from non-expressing ones, an analysis of the transcription of individual copies is required, which is difficult to implement at this stage due to the short length and high homology of coding 5S rDNA regions.

Thus, using pyrosequencing and subsequent computer analysis of three BAC clones bearing the genes of 5S rRNA, we first reconstructed the two extended regions on the 5BS chromosome of bread wheat containing these genes and revealed their different patterns of structural organization.

## Conclusions

The extended regions bearing 5S rRNA genes located on chromosome 5BS of bread wheat were first described in this work. Sequencing and subsequent analysis of these regions showed that they differ in their structural organization, one of them presents a highly diverged type of organization with multiple insertions of TE interrupting the 5S rDNA arrays, while the another is a single cluster of units. Also, these regions differ in the origin and level of heterogeneity of units: the first includes short units, variable in their spacers that are related to ShortA1 and ShortA2 haplomes, whereas the second consists of uniform long units of LongS1 haplome. Another interesting finding is the absence of FISH signal from the short unit probe in chromosomes of A- and B- diploid precursors of *T. aestivum,* pointing to a high divergence of these units since the formation of polyploid wheats.

## Additional files

Additional file 1: Table S1. The description of 5S rDNA-tagged genomic fragments from 5BS chromosome of the bread wheat. The fragments are presented as the contigs resulting from shotgun 454-sequencing (for pools 52 and 89) and as a scaffolds resulting from paired-ends 454-sequencing (pool_52). The presence of complete homology with BAC-end sequences is indicated. Moreover some of fragments were additionally confirmed and elongated with the data obtained from IonTorrent BAC-sequencing and PCR sequencing with specific primers (J2f2 5′- AGGTGTTACCAGCTAGATCGATGTGACATC-3′ and 010 L1 5′-AGAGGCCCTTATCTATTTCCAGAATTGCTG-3′). (DOC 39 kb)

Additional file 2: Figure S1. The PCR testing for the overlapping of BAC-clones of pool_52 (TaaCsp5BS025F09 and TaaCsp5BS010O13). The positions of BAC End Sequences for two fragments derived from TaaCsp5BS025F09 indicated by red arrows. The three pairs of ISBP primers were designed to TE insertions (Danae-1/Fatima: 5′-GACAAAAATGGCCAACATCC-3′ and 5′- GACCCCCTAATCCAGGACTC-3′; Fatima/Danae-1: 5′-TGTCCCCAGCCTCTTGTTAC-3′ and 5′- GTGAAGGTGCCAACGAACTC-3′; 5SrDNA/Laura: 5′-ACCCTAGTTGGTTTCAGAGG-3′ and 5′- TGGGTGCTCACGATTCAC-3′) are indicated by black arrows. Primers were tested on the individual BAC-DNA templates TaaCsp5BS025F09 and TaaCsp5BS010O13. The PCR amplification results for each primer pairs presented over the corresponding TE junction and demonstrate the overlapping of TaaCsp5BS025F09 and TaaCsp5BS010O13. (DOCX 152 kb)

Additional file 3: Table S2. The dataset obtained from shotgun+paired reads sequencing of pool 52. (DOCX 12 kb)

Additional file 4: The MUSCLE alignment of pool_52 5S rDNA coding sequences (a), and pool_89 5S rDNA coding sequences (b). The corresponding read number for each representative sequence denoted. (DOCX 20 kb)

Additional file 5: Cluster analysis of non transcribed spacers of 5S rDNA for pool_89 (A) and pool_52 (B) sequences. (DOCX 17 kb)

Additional file 6: The MUSCLE Alignment of representative 5S rDNA spacers from each cluster of pool_89 and LongS1 unit type [10]. The number of spacer sequences in cluster indicated in brackets. The conserved sites indicated as asterisk. The position of Long89_F and Long89_R primers used to obtain the FISH probe indicated by grey colour. (DOCX 13 kb)

Additional file 7: The MUSCLE Alignment of representative 5S rDNA spacers from each tree brunch of pool_52 with ShortA1, ShortA2 and ShortG1 types of 5S rDNA units [10]. The number of spacer sequences in cluster indicated in brackets. The conserved sites indicated as asterisk. The position of Short52_F and Short52_R primers used to obtain the FISH probe indicated by grey colour. (DOCX 13 kb)

## Abbreviations

BAC: Bacterial artificial chromosome
BES: BAC-end sequence
BLAST: Basic local alignment search tool
DTC: CACTA DNA-transposon
FISH: Fluorescence in situ hybridization
ISBP: Insertion size based polymorphism
LTR: Long terminal repeat
NTS: Non-transcribed spacer
rDNA: Ribosomal DNA
RLG: Gypsy LTR-retrotransposon
TE: Transposable element
