# Supplementary material for: Fine organization of genomic regions tagged to the 5S rDNA locus of the bread wheat 5B chromosome
Source: BMC Plant Biol. 2017 Nov 14;17(Suppl 1):183. doi: 10.1186/s12870-017-1120-5 (PMC5688495; doi:10.1186/s12870-017-1120-5)
Supplement: Supplementary file 1 — The description of 5S rDNA-tagged genomic fragments from 5BS chromosome of the bread wheat. The fragments are presented as the contigs resulting from shotgun 454-sequencing (for pools 52 and 89) and as a scaffolds resulting from paired-ends 454-sequencing (pool_52). The presence of complete homology with BAC-end sequences is indicated. Moreover some of fragments were additionally confirmed and elongated with the data obtained from IonTorrent BAC-sequencing and PCR sequencing with specific primers (J2f2 5′- AGGTGTTACCAGCTAGATCGATGTGACATC-3′ and 010 L1 5′-AGAGGCCCTTATCTATTTCCAGAATTGCTG-3′). (DOC 39 kb) [file 12870_2017_1120_MOESM1_ESM.doc]

| **Construction method** | | | | | | |
| --- | --- | --- | --- | --- | --- | --- |
| **Pool_52** | | | | | | |
|  | **Length, bp** | **Paired-end 454 sequencing** | **454 sequencing** | **BAC-End-Sequencing (BES)** | **IonTorrent** | **PCR with specific primers** |
| **5S rDNA Fragment 1** | 10801 | 5’-fragment (10801 bp) of Scf_001 (164054 bp) | Ctg_0017 (4058 bp) | 025F09_M13  (961 bp) | n/a | n/a |
| **5S rDNA Fragment 2** | 52840 | Scf_003 (52487 bp) | Ctg_0003 (52492 bp) | 025F09_T7  (807 bp) | BAC_c571 (17042 bp) | n/a |
| **5S rDNA Fragment 3** | 39574 | Scf_004 (37321 bp) | Ctg_0007 (9537 bp)  Ctg_0010 (8642 bp)  Ctg_0008 (9409 bp)  Ctg_0013 (5329 bp) | - | n/a | j2f2/010L1 (819 bp) |
| **5S rDNA Fragment 4** | 30284 | 5’-fragment (30284 bp) of Scf _005 (32106 bp) | Ctg_0005 (27852 bp) | 010O13_M13  (498 bp) | n/a | n/a |
| **5S rDNA Fragment 5** | 4415 | Scf_006 (4365 bp) | Ctg_0015 (4415 bp) | 010O13_T7  (736 bp) | n/a | n/a |
| **Pool_89** | | | | | | |
| **5S rDNA Fragment 6** | 2503 | - | Ctg_0069 (2415 bp) | n/a | BAC_rep_c5604 (1040 bp) | n/a |

Additional_File 1: Table S1. The description of 5S rDNA-tagged genomic fragments from 5BS chromosome of the bread wheat. The fragments are presented as the contigs resulting from shotgun 454-sequencing (for pools 52 and 89) and as a scaffolds resulting from paired-ends 454-sequencing (pool_52). The presence of complete homology with BAC-end sequences is indicated. Moreover some of fragments were additionally confirmed and elongated with the data obtained from IonTorrent BAC-sequencing and PCR sequencing with specific primers (J2f2 5’- AGGTGTTACCAGCTAGATCGATGTGACATC-3’ and 010L1 5’-AGAGGCCCTTATCTATTTCCAGAATTGCTG-3’)
