# Supplementary material for: Fine organization of genomic regions tagged to the 5S rDNA locus of the bread wheat 5B chromosome
Source: BMC Plant Biol. 2017 Nov 14;17(Suppl 1):183. doi: 10.1186/s12870-017-1120-5 (PMC5688495; doi:10.1186/s12870-017-1120-5)
Supplement: Supplementary file 2 — The PCR testing for the overlapping of BAC-clones of pool_52 (TaaCsp5BS025F09 and TaaCsp5BS010O13). The positions of BAC End Sequences for two fragments derived from TaaCsp5BS025F09 indicated by red arrows. The three pairs of ISBP primers were designed to TE insertions (Danae-1/Fatima: 5′-GACAAAAATGGCCAACATCC-3′ and 5′- GACCCCCTAATCCAGGACTC-3′; Fatima/Danae-1: 5′-TGTCCCCAGCCTCTTGTTAC-3′ and 5′- GTGAAGGTGCCAACGAACTC-3′; 5SrDNA/Laura: 5′-ACCCTAGTTGGTTTCAGAGG-3′ and 5′- TGGGTGCTCACGATTCAC-3′) are indicated by black arrows. Primers were tested on the individual BAC-DNA templates TaaCsp5BS025F09 and TaaCsp5BS010O13. The PCR amplification results for each primer pairs presented over the corresponding TE junction and demonstrate the overlapping of TaaCsp5BS025F09 and TaaCsp5BS010O13. (DOCX 152 kb) [file 12870_2017_1120_MOESM2_ESM.docx]

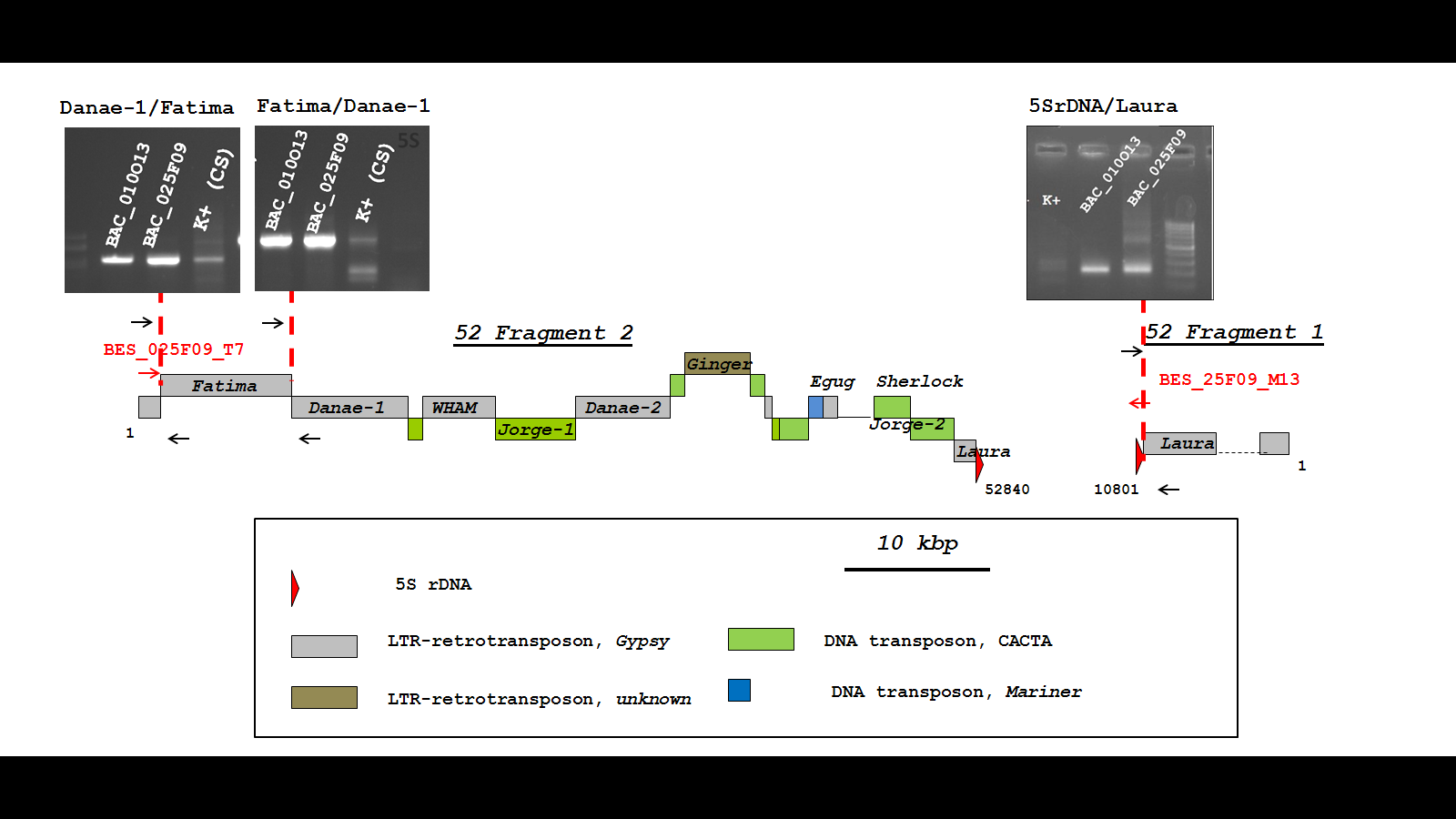


**Additional File 2: Figure S1. The PCR testing for the overlapping of BAC-clones of pool_52** (**TaaCsp5BS025F09 and TaaCsp5BS010O13)**. The positions of BAC End Sequences for two fragments derived from TaaCsp5BS025F09 indicated by red arrows. The three pairs of ISBP primers were designed to TE insertions (Danae-1/Fatima: 5’-GACAAAAATGGCCAACATCC-3’ and 5’- GACCCCCTAATCCAGGACTC-3’; Fatima/Danae-1: 5’-TGTCCCCAGCCTCTTGTTAC-3’ and 5’- GTGAAGGTGCCAACGAACTC-3’; 5SrDNA/Laura: 5’-ACCCTAGTTGGTTTCAGAGG-3’ and 5’- TGGGTGCTCACGATTCAC-3’) are indicated by black arrows. Primers were tested on the individual BAC-DNA templates TaaCsp5BS025F09 and TaaCsp5BS010O13. The PCR amplification results for each primer pairs presented over the corresponding TE junction and demonstrate the overlapping of TaaCsp5BS025F09 and TaaCsp5BS010O13.
