# Supplementary material for: Fine organization of genomic regions tagged to the 5S rDNA locus of the bread wheat 5B chromosome
Source: BMC Plant Biol. 2017 Nov 14;17(Suppl 1):183. doi: 10.1186/s12870-017-1120-5 (PMC5688495; doi:10.1186/s12870-017-1120-5)
Supplement: Supplementary file 3 — The dataset obtained from shotgun+paired reads sequencing of pool 52. (DOCX 12 kb) [file 12870_2017_1120_MOESM3_ESM.docx]

**Additional File 3: Table S2.** The dataset obtained from shotgun+paired reads sequencing of pool 52

|  | Pool_52:  6 BAC-clones | Pool_89:  10 BAC-clones |
| --- | --- | --- |
| 5S rDNA-tagged clones | TaaCsp5BS010O13  TaaCsp5BS025F09 | TaaCsp5BS096G09 |
| Number of bases, bp | 70991 | 65190 |
| Number of reads | 24376938 | 20504835 |
| Number of paired end reads | 10804 | 9851 |
| Number of scaffolds | 11 | 10 |
| Length of scaffolds, bp | 445788 | 684953 |
| N50 of scaffolds, bp | 137010 | 199581 |
| Largest scaffold, bp | 164054 | 202599 |
| Average scaffold size | 40526 | 68495 |
