# Supplementary material for: Fine organization of genomic regions tagged to the 5S rDNA locus of the bread wheat 5B chromosome
Source: BMC Plant Biol. 2017 Nov 14;17(Suppl 1):183. doi: 10.1186/s12870-017-1120-5 (PMC5688495; doi:10.1186/s12870-017-1120-5)
Supplement: Supplementary file 4 — The MUSCLE alignment of pool_52 5S rDNA coding sequences (a), and pool_89 5S rDNA coding sequences (b). The corresponding read number for each representative sequence denoted. (DOCX 20 kb) [file 12870_2017_1120_MOESM4_ESM.docx]

**Additional File 4**. **The MUSCLE alignment of pool_52 5S rDNA coding sequences.** (a), and pool_89 5S rDNA coding sequences (b). The corresponding read number for each representative sequence denoted.

A.

JMOB0DO02GFBRZ_0120(+)_5 GGATGCGATCATACCAGCACTAAAGCAtCGGATttCATtAG-AACTCCa-AAGTTAAGCG

JMOB0DO02GNKJL_112234()_9 GGATGCGATCATgCCAGCACTAAAGCACtGGATCCCATCAG-AACTCCG-AAGTTAAGCG

JMOB0DO02G7J5F_21141()_9 aGATGCGATtATACCAGCACTAAAGCAtCGGATCCCATCAa-AACTCtG-AAGTTAAGCG

JMOB0DO02H7FW6_22144(+)_5 tGATGCGATCATACCAGCACTAAAGCACCGGATCCCATCAG-AACTCtG-AAGTTAAGCG

JMOB0DO02H8L4Z_50172(+)_7 GGATtgGATCATACCAGCACTAAAGCACCGGATCCCATCAG-AACTtCG-AAGTTAAGCG

JMOB0DO02ICC0B_0122(+)_14 aGATGCGATCATACCAGCACTAAAGCACCGGATCCCATCAG-AACTCCG-AAGTTAAGCG

JMOB0DO02H436Z_28150(+)_10 GGAaGCGATCATACCAGCACTAAAGCACCGGATCCCATCAG-AACTCCG-AAaTTAAGCG

JMOB0DO02F7ER6_177299()_7 GGATGCGATCATACCAGCACTAAAGCAtCGGATCCCATtAG-AACTCCG-AAGTTAAGCG

JMOB0DO02FLGF5_199319(+)_5 GGATGCGATCATACCAGCACTAAAGCACCGGATCCCATCAG-AACTCCG-AAGTTAAGCG

JMOB0DO02IFSRZ_62183()_11 GGATGCGATCATACCAGCACTAAAGCACCGGATCCCATCAG-AACTCCG-AAGTTAAGCG

JMOB0DO02IW27G_160283(+)_12 GGATGCGATCATACCAGCACTAAAGCACCGGATCCCATCAG-AACTCCG-AAGTTAAGCG

JMOB0DO02GN8YS_240360(+)_10 GGATGCGATCATACCAGCACTAAAGCACCGGATCCCATCAG-AACTCCG-AAGTTAAGCG

JMOB0DO02GNZVS_209331()_9 GGATGCGATCATACCAGCACTAAAGCACCGGATCCCATCAG-AACTCCG-AAGTTAAGCG

JMOB0DO02HUDG4_48170(+)_29 GGATGCGATCATACCAGCACTAAAGCACCGGATCCCATCAG-AACTCCG-AAGTTAAGCG

JMOB0DO02I696V_71193()_5 GGATGCGATCATACCAGCACTAAAGCACCGGATCCCATCAG-AACTCCG-AAGTTAAGCG

JMOB0DO02GH9UB_95217(+)_5 GGATGCGATCATACCAGCACTAAAGCACCGGATCCCATCAG-AAaTCCG-AAGTTAAGCG

JMOB0DO02ID7TQ_88210(+)_16 GGATGCGATCATACCAGCACTAAAGCACCGGATCCCATCAG-AACTCCG-AAGTTAAGtG

JMOB0DO02HI4NI_26148(+)_13 GGATGCGgTCATACCAGCACTAAAGCACCGGATCCCATCAG-AACTCCG-AAGTTAAGCG

JMOB0DO02IJO1M_0122(+)_13 GGATGtGATCATACCAGCACTAAAGCACCGGATCCCATCAG-AACTCCG-AAGTTAAGCG

JMOB0DO02HJXNY_102224()_11 GGATGCGATCATACCAGCACTAAAGCACCGGATCCCATCAG-AACTCtG-AAGTTAAGCG

JMOB0DO02IQ322_25147()_7 GGATGCGATCATACCAGCACTAAAGCACaGGATCCCATCAG-AACTCCG-AAGTTAAGCG

JMOB0DO02G0MII_10132(+)_7 GGATGCGATCATACCAGCACTAAAGCACCGGATCCCATCAG-AACTCCG-AAGTTAAcCG

JMOB0DO02F6PZK_72195(+)_7 GGATGCGATCATACCAGCACTAAAGCACCGGATCCCATCAG-AACTCCGtAAGTTAAGCG

JMOB0DO02HTYPF_242363()_5 GGATGCGATCATA-CAGCACTAAAGCACCGGATCCCATCAG-AACTCCG-AAGTTAAGCG

JMOB0DO02G9ECO_126247()_6 GGATGCGATCATACCAGCACTAAAGCACCGGATCCCATCAG-AACTCCG-AAGTTAAGCG

JMOB0DO02GNWTH_288409()_8 GGATGCGATCATACCAGCACTAAAGCACCGGATCCCATCAG-AACTCCG-AAGTTAAGCG

JMOB0DO02IUL0N_88209()_15 GGATGCGATCATACCAGCACTAAAGCACCGGAT-CCATCAG-AACTCCG-AAGTTAAGCG

JMOB0DO02HAPW1_83206()_6 GGATGCGATCATACCAGCACTAAAGCACCGGATCCCATCAGaAACTCCG-AAGTTAAGCG

JMOB0DO02HAW8D_98221()_7 GGATGCGATCATACCAGCACTAAAGCACCGGATCCCATCAG-AACTCCG-AAGTTAAGCG

JMOB0DO02GYIDF_35157()_700 GGATGCGATCATACCAGCACTAAAGCACCGGATCCCATCAG-AACTCCG-AAGTTAAGCG

JMOB0DO02GFBRZ_0120(+)_5 TGCTTGGGCGAGAGTAGTACTAGGATGGGTGA--CtTCta-GGGAAGTCCTCcaG-TTGC

JMOB0DO02GNKJL_112234()_9 TcCTTGGGtGAGAGTAGTACTAGGATGGGTGAccCCcCCT-acGAAGTCCTCGTG-TTGC

JMOB0DO02G7J5F_21141()_9 TGCTTGGGCGAGAGTAGTACTAGGATGGcTGA--CCTCCT-GGGAAGTCCTCGTG-TTGC

JMOB0DO02H7FW6_22144(+)_5 TGCTTGGGtGAGAtTAGTACTAGGATGGGTGA--CCTCCT-GGGAAGTCCTCGTG-TTGC

JMOB0DO02H8L4Z_50172(+)_7 TGCTTGGGCGAGAGTAGTACTAGGATGGGTGA--CCTCCT-GGGAAGTCCTCGTG-TTGC

JMOB0DO02ICC0B_0122(+)_14 TGCTTGGGCGAGAGTAGTACTAGGATGGaTGA--CCTCCT-GGGAAGTCCTCaTG-TTGC

JMOB0DO02H436Z_28150(+)_10 TGCTTGGGCGAGAGTAGTACTAGGATGGGTGA--CCTCCT-GGGAAGTCCTCGTG-TTGC

JMOB0DO02F7ER6_177299()_7 TGCTTGGGCGAGAGTAGTACTAGGATGGGTGA--CCTCCT-GGGAAGTCCTCGTG-TTGC

JMOB0DO02FLGF5_199319(+)_5 TGCTTGGGCGAGAGTAGTACTAGGATGGGTGA--CCTCCT-GGGAAGTCCTCGTGtTTGC

JMOB0DO02IFSRZ_62183()_11 TGCTTGGGCGAGAGTAGTACTAGGATGGGTGA--CCTCCT-GGGAAGTCCTCGTG-TTGC

JMOB0DO02IW27G_160283(+)_12 TGCTTGGGCGAGAGTAGTACTAGGATGGGTGA--CCTCCT-GGGAAGTCCTCGTG-TTGC

JMOB0DO02GN8YS_240360(+)_10 TGCTTGGGCGAGAGTAGTACTAGGATGGGTGA--CCTCCT-GGGAAGTCCTCGTG-TTGC

JMOB0DO02GNZVS_209331()_9 TGCTTGGGCGAGAGTAGTACTAGGATGGGTGA--CCTCCT-GGGAAGTCCTCGTG-TTGC

JMOB0DO02HUDG4_48170(+)_29 TGCTTGGGCGAGAGTAGTACTAGGATGGGTGA--CCTCCT-GGGAAGTCCTCGTG-TTGC

JMOB0DO02I696V_71193()_5 TGCTTGGGCGAGAGTAGTACTAGGATGGGTGA--CCTCCT-GGGAAGTCtTCGTG-TTGC

JMOB0DO02GH9UB_95217(+)_5 TGCTTGGGCGAGAGTAGTACTAGGATGGGTGA--CCTCCT-GGGAAGTCCTCGTG-TTGC

JMOB0DO02ID7TQ_88210(+)_16 TGCTTGGGCGAGAGTAGTACTAGGATGGGTGA--CCTCCT-GGGAAGTCCTCGTG-TTGC

JMOB0DO02HI4NI_26148(+)_13 TGCTTGGGCGAGAGTAGTACTAGGATGGGTGA--CCTCCT-GGGAAGTCCTCGTG-TTGC

JMOB0DO02IJO1M_0122(+)_13 TGCTTGGGCGAGAGTAGTACTAGGATGGGTGA--CCTCCT-GGGAAGTCCTCGTG-TTGC

JMOB0DO02HJXNY_102224()_11 TGCTTGGGCGAGAGTAGTACTAGGATGGGTGA--CCTCCT-GGGAAGTCCTCGTG-TTGC

JMOB0DO02IQ322_25147()_7 TGCTTGGGCGAGAGTAGTACTAGGATGGGTGA--CCTCCT-GGGAAGTCCTCGTG-TTGC

JMOB0DO02G0MII_10132(+)_7 TGCTTGGGCGAGAGTAGTACTAGGATGGGTGA--CCTCCT-GGGAAGTCCTCGTG-TTGC

JMOB0DO02F6PZK_72195(+)_7 TGCTTGGGCGAGAGTAGTACTAGGATGGGTGA--CCTCCT-GGGAAGTCCTCGTG-TTGC

JMOB0DO02HTYPF_242363()_5 TGCTTGGGCGAGAGTAGTACTAGGATGGGTGA--CCTCCT-GGGAAGTCCTCGTG-TTGC

JMOB0DO02G9ECO_126247()_6 TGCTTGGGCGAGAGTAGTACTAGGATGGGTGA--CCTCCT-GGGAAGTC-TCGTG-TTGC

JMOB0DO02GNWTH_288409()_8 TGCTTGGGCGAGAGTAGTACTAGGATGGGTGA--CCTCCT-GGGAAGTCCTCGTG--TGC

JMOB0DO02IUL0N_88209()_15 TGCTTGGGCGAGAGTAGTACTAGGATGGGTGA--CCTCCT-GGGAAGTCCTCGTG-TTGC

JMOB0DO02HAPW1_83206()_6 TGCTTGGGCGAGAGTAGTACTAGGATGGGTGA--CCTCCT-GGGAAGTCCTCGTG-TTGC

JMOB0DO02HAW8D_98221()_7 TGCTTGGGCGAGAGTAGTACTAGGATGGGTGA--CCTCCTgGGGAAGTCCTCGTG-TTGC

JMOB0DO02GYIDF_35157()_700 TGCTTGGGCGAGAGTAGTACTAGGATGGGTGA--CCTCCT-GGGAAGTCCTCGTG-TTGC

JMOB0DO02GFBRZ_0120(+)_5 ATTCCC---

JMOB0DO02GNKJL_112234()_9 ATTCCC---

JMOB0DO02G7J5F_21141()_9 ATTCCC---

JMOB0DO02H7FW6_22144(+)_5 ATTCCCcT-

JMOB0DO02H8L4Z_50172(+)_7 ATTCCCTT-

JMOB0DO02ICC0B_0122(+)_14 ATTCCCTT-

JMOB0DO02H436Z_28150(+)_10 ATTCCCTT-

JMOB0DO02F7ER6_177299()_7 ATTCCCTT-

JMOB0DO02FLGF5_199319(+)_5 ATcCC----

JMOB0DO02IFSRZ_62183()_11 ATcCCtT--

JMOB0DO02IW27G_160283(+)_12 ATTtCCTTt

JMOB0DO02GN8YS_240360(+)_10 ATTtCC---

JMOB0DO02GNZVS_209331()_9 ATTCCCcT-

JMOB0DO02HUDG4_48170(+)_29 ATTCCtTT-

JMOB0DO02I696V_71193()_5 ATTCCCTT-

JMOB0DO02GH9UB_95217(+)_5 ATTCCCTT-

JMOB0DO02ID7TQ_88210(+)_16 ATTCCCTT-

JMOB0DO02HI4NI_26148(+)_13 ATTCCCTT-

JMOB0DO02IJO1M_0122(+)_13 ATTCCCTT-

JMOB0DO02HJXNY_102224()_11 ATTCCCTT-

JMOB0DO02IQ322_25147()_7 ATTCCCTT-

JMOB0DO02G0MII_10132(+)_7 ATTCCCTT-

JMOB0DO02F6PZK_72195(+)_7 ATTCCCTT-

JMOB0DO02HTYPF_242363()_5 ATTCCCTT-

JMOB0DO02G9ECO_126247()_6 ATTCCCTT-

JMOB0DO02GNWTH_288409()_8 ATTCCCTT-

JMOB0DO02IUL0N_88209()_15 ATTCCCTT-

JMOB0DO02HAPW1_83206()_6 ATTCCCTT-

JMOB0DO02HAW8D_98221()_7 ATTCCCTT-

JMOB0DO02GYIDF_35157()_700 ATTCCCTT-

B.

JXUJKF302HSIHL_13136()_5 GGATGCGATCATACCagaCACTAAAGCACCGGATCCCATCAG-AACTCCGAAGTTAAGCG

JXUJKF302F5NBW_92212()_5 GGATGCGATCATA-CCAGCACTAAAGCAtCGGATCCCATCAG-AACTCCGAAGTTAAGCG

JXUJKF302HYXKK_11133()_7 GGATGCGATCATA-CCAGCACTAAAGCACCGGATCCCATCAG-AACTCCGAAGTTAAGCG

JXUJKF302HKGAU_164285()_5 GGATGCGATCATA-CCAGCACTAAAGCACCGGATCCCATCAG-AACTCCGAAGTTAAGCG

JXUJKF302FPETS_70191(+)_8 GGATGCGATCATA-CaAGCA-TAAAGCACCGGATCCCATCAG-AACTCCGAAGTTAAGCG

JXUJKF302FM1KC_27149(+)_25 GGATGCGATCATA-CaAGCACTAAAGCACCGGATCCCATCAG-AACTCCGAAGTTAAGCG

JXUJKF302JGAW6_91214()_6 GGATGCGATCATACCaAGCACTAAAGCACCGGATCCCATCAG-AACTCCGAAGTTAAGCG

JXUJKF302G1OHU_10132(+)_7 GGATGCGAgCATA-CCAGCACTAAAGCACCGGATCCCATCAG-AACTCCGAAGTTAAGCG

JXUJKF302G1R00_0122(+)_6 GGATGCGATCATA-CCAGtACTAAAGCACCGGATCCCATCAG-AACTCCGAAGTTAAGCG

JXUJKF302GBU7E_21143()_5 GGATGCGATCATA-CCAGCACTAAAGCACCGGATCCCATCAG-AACTCCGAAGTTAAGCG

JXUJKF302HB7GP_13135()_5 GGATGCGATCATA-CCAGCACTAAAGCACCGGATCCCATCAG-AACTCCGAAGTTAAGCG

JXUJKF302HFQW7_48170()_7 GGATGCGATCATA-CCAGCACTAAAGCACCGGATCCCATCAG-AACTtCGAAGTTAAGCG

JXUJKF302GNZQS_122243()_12 GGATGCGATCATA-CCAGCACTAAAGCACCGGATCCCATCAG-AACT-CGAAGTTAAGCG

JXUJKF302F87FD_59181()_5 GGATGCGATCATA-CCAGCACTAAAGCAtCGGATCCCATCAG-AACTCCGAAGTTAAGCG

JXUJKF302GMJ58_48169()_5 GGATGCGATCATA-CCAGCACTAAAGCA-CGGATCCCATCAG-AACTCCGAAGTTAAGCG

JXUJKF302HKC47_103226()_6 GGATGCGATCATA-CCAGCACTAAAGCACCGGATCCCATCAGaAACTCCGAAGTTAAGCG

JXUJKF302H0OZS_104226(+)_170 GGATGCGATCATA-CCAGCACTAAAGCACCGGATCCCATCAG-AACTCCGAAGTTAAGCG

JXUJKF302H69XP_46167()_13 GGATGCGATCATA-CCAGCACTAAAGCACCGGATCCCAT-AG-AACTCCGAAGTTAAGCG

JXUJKF302IJTZ6_65186()_8 GGATGCGATCATA-CCAGCACTAAAGCACCGGATCCCATCAG-AACTCCGAAGTTAAGCG

JXUJKF302GJ99E_31152(+)_10 GGATGCGATCATA-CCAGCACTAAAGCACCGGATCCCATCAG-AACTCCGAAGTTAAGCG

JXUJKF302GFFR0_32154(+)_6 GGATGCGATCATA-CCAGCACTAAAGCACCGGATCCCATCAG-AACTCCGAAGTTAAGCG

JXUJKF302HSIHL_13136()_5 TGCTTGGGCGAGAGTAGTACTAGGATGGGT-GACCTCCTGGGAAGTCCTCGTGTTGCATT

JXUJKF302F5NBW_92212()_5 TGCTTGGGCGAGAGTAGTACTAGGATGGGT-GACCTCCTGGGAAGTCCTCGTGTTGCATT

JXUJKF302HYXKK_11133()_7 TGCTTGGGCGAGAGTAGTACTAGGATGGGT-GAaCTCCTGGGAAGTCCTCGTGTTGCATT

JXUJKF302HKGAU_164285()_5 TGCTTGGGCGAGAGTAGTACTAGGATGGGT-GA-CTCCTGGGAAGTCCTCGTGTTGCATT

JXUJKF302FPETS_70191(+)_8 TGCTTGGGCGAGAGTAGTACTAGGATGGGT-GACCTCCTGGGAAGTCCTCGTGTTGCATT

JXUJKF302FM1KC_27149(+)_25 TGCTTGGGCGAGAGTAGTACTAGGATGGGT-GACCTCCTGGGAAGTCCTCGTGTTGCATT

JXUJKF302JGAW6_91214()_6 TGCTTGGGCGAGAGTAGTACTAGGATGGGT-GACCTCCTGGGAAGTCCTCGTGTTGCATT

JXUJKF302G1OHU_10132(+)_7 TGCTTGGGCGAGAGTAGTACTAGGATGGGT-GACCTCCTGGGAAGTCCTCGTGTTGCATT

JXUJKF302G1R00_0122(+)_6 TGCTTGGGCGAGAGTAGTACTAGGATGGGT-GACCTCCTGGGAAGTCCTCGTGTTGCATT

JXUJKF302GBU7E_21143()_5 TGCTTGGGCGtGAGTAGTACTAGGATGGGT-GACCTCCTGGGAAGTCCTCGTGTTGCATT

JXUJKF302HB7GP_13135()_5 TGCTTGGGCGAGAGTAGTACTAGGATGGGc-GACCTCCTGGGAAGTCCTCGTGTTGCATT

JXUJKF302HFQW7_48170()_7 TGCTTGGGCGAGAGTAGTACTAGGATGGGT-GACCTCCTGGGAAGTCCTCGTGTTGCATT

JXUJKF302GNZQS_122243()_12 TGCTTGGGCGAGAGTAGTACTAGGATGGGT-GACCTCCTGGGAAGTCCTCGTGTTGCATT

JXUJKF302F87FD_59181()_5 TGCTTGGGCGAGAGTAGTACTAGGATGGGT-GACCTCCTGGGAAGTCCTCGTGTTGCATT

JXUJKF302GMJ58_48169()_5 TGCTTGGGCGAGAGTAGTACTAGGATGGGT-GACCTCCTGGGAAGTCCTCGTGTTGCATT

JXUJKF302HKC47_103226()_6 TGCTTGGGCGAGAGTAGTACTAGGATGGGT-GACCTCCTGGGAAGTCCTCGTGTTGCATT

JXUJKF302H0OZS_104226(+)_170 TGCTTGGGCGAGAGTAGTACTAGGATGGGT-GACCTCCTGGGAAGTCCTCGTGTTGCATT

JXUJKF302H69XP_46167()_13 TGCTTGGGCGAGAGTAGTACTAGGATGGGT-GACCTCCTGGGAAGTCCTCGTGTTGCATT

JXUJKF302IJTZ6_65186()_8 TGCTTGGGCGAGAGTAGTACTAGGATGGGTgGACCTCCTGGGAAtTCCTCGTG-TGCA-T

JXUJKF302GJ99E_31152(+)_10 TGCTTGGGCGAGAGTAGTACTAGGATGGGT-GACCTCCTGGGAA-TCCTCGTGTTGCATT

JXUJKF302GFFR0_32154(+)_6 TGCTTGGGCGAGAGTAGTACTAGGATGGGT-GACCTCCTGGGAAtTCCTCGTGTTGCATT

JXUJKF302HSIHL_13136()_5 CCTTT

JXUJKF302F5NBW_92212()_5 CtT--

JXUJKF302HYXKK_11133()_7 CCTTT

JXUJKF302HKGAU_164285()_5 CCTTT

JXUJKF302FPETS_70191(+)_8 CCTTT

JXUJKF302FM1KC_27149(+)_25 CCTTT

JXUJKF302JGAW6_91214()_6 CCTTT

JXUJKF302G1OHU_10132(+)_7 CCTTT

JXUJKF302G1R00_0122(+)_6 CCTTT

JXUJKF302GBU7E_21143()_5 CCTTT

JXUJKF302HB7GP_13135()_5 CCTTT

JXUJKF302HFQW7_48170()_7 CCTTT

JXUJKF302GNZQS_122243()_12 CCTTT

JXUJKF302F87FD_59181()_5 CCTTT

JXUJKF302GMJ58_48169()_5 CCTTT

JXUJKF302HKC47_103226()_6 CCTTT

JXUJKF302H0OZS_104226(+)_170 CCTTT

JXUJKF302H69XP_46167()_13 CCTTT

JXUJKF302IJTZ6_65186()_8 CCTTT

JXUJKF302GJ99E_31152(+)_10 CCTTT

JXUJKF302GFFR0_32154(+)_6 CCTTT
