# Supplementary material for: Fine organization of genomic regions tagged to the 5S rDNA locus of the bread wheat 5B chromosome
Source: BMC Plant Biol. 2017 Nov 14;17(Suppl 1):183. doi: 10.1186/s12870-017-1120-5 (PMC5688495; doi:10.1186/s12870-017-1120-5)
Supplement: Supplementary file 5 — Cluster analysis of non transcribed spacers of 5S rDNA for pool_89 (A) and pool_52 (B) sequences. (DOCX 17 kb) [file 12870_2017_1120_MOESM5_ESM.docx]

**Additional File 5.** Cluster analysis of non transcribed spacers of 5S rDNA for pool_89(A) and pool_52 (B) sequences.

**A.**

>Cluster 0

0 367bp, >spacer_1... at 98.91%

1 370bp, >spacer_2... *

2 369bp, >spacer_6... at 98.64%

3 367bp, >spacer_7... at 99.18%

4 368bp, >spacer_8... at 98.37%

5 366bp, >spacer_9... at 98.09%

6 369bp, >spacer_11... at 98.64%

7 369bp, >spacer_16... at 98.37%

8 367bp, >spacer_17... at 98.91%

9 367bp, >spacer_20... at 98.91%

10 368bp, >spacer_21... at 98.64%

11 367bp, >spacer_22... at 98.91%

12 369bp, >spacer_25... at 98.37%

13 367bp, >spacer_26... at 98.91%

14 366bp, >spacer_27... at 98.91%

>Cluster 1

0 368bp, >spacer_3... at 100.00%

1 369bp, >spacer_4... at 99.19%

2 369bp, >spacer_5... at 99.73%

3 368bp, >spacer_10... at 100.00%

4 369bp, >spacer_12... at 100.00%

5 369bp, >spacer_13... at 98.37%

6 368bp, >spacer_15... at 100.00%

7 370bp, >spacer_18... *

8 369bp, >spacer_19... at 98.37%

9 367bp, >spacer_23... at 99.73%

>Cluster 2

0 368bp, >spacer_14... *

1 366bp, >spacer_24... at 99.18%

**B.**

>Cluster 0

0 390bp, >spacer_110... *

>Cluster 1

0 343bp, >spacer_15... *

>Cluster 2

0 339bp, >spacer_91... *

>Cluster 3

0 338bp, >spacer_38... *

>Cluster 4

0 337bp, >spacer_79... *

>Cluster 5

0 336bp, >spacer_73... *

>Cluster 6

0 334bp, >spacer_105... *

>Cluster 7

0 332bp, >spacer_23... *

1 326bp, >spacer_28... at 99.08%

>Cluster 8

0 331bp, >spacer_58... *

>Cluster 9

0 330bp, >spacer_20... *

1 329bp, >spacer_68... at 100.00%

>Cluster 10

0 330bp, >spacer_35... *

>Cluster 11

0 329bp, >spacer_3... *

>Cluster 12

0 329bp, >spacer_90... *

>Cluster 13

0 328bp, >spacer_5... *

>Cluster 14

0 328bp, >spacer_9... *

>Cluster 15

0 328bp, >spacer_96... *

>Cluster 16

0 327bp, >spacer_29... *

>Cluster 17

0 327bp, >spacer_82... *

>Cluster 18

0 326bp, >spacer_24... *

>Cluster 19

0 325bp, >spacer_6... *

>Cluster 20

0 325bp, >spacer_21... *

>Cluster 21

0 325bp, >spacer_133... *

>Cluster 22

0 324bp, >spacer_34... *

>Cluster 23

0 323bp, >spacer_47... *

>Cluster 24

0 323bp, >spacer_63... *

>Cluster 25

0 318bp, >spacer_19... *

>Cluster 26

0 315bp, >spacer_59... *

>Cluster 27

0 315bp, >spacer_60... *

>Cluster 28

0 315bp, >spacer_108... *

>Cluster 29

0 297bp, >spacer_1... at 98.99%

1 294bp, >spacer_2... at 99.32%

2 287bp, >spacer_4... at 98.26%

3 294bp, >spacer_8... at 98.30%

4 298bp, >spacer_11... at 98.32%

5 294bp, >spacer_12... at 98.64%

6 295bp, >spacer_13... at 99.32%

7 294bp, >spacer_16... at 98.30%

8 296bp, >spacer_22... at 98.65%

9 297bp, >spacer_25... at 99.66%

10 298bp, >spacer_26... at 98.32%

11 295bp, >spacer_27... at 98.98%

12 296bp, >spacer_30... at 98.31%

13 294bp, >spacer_31... at 99.32%

14 295bp, >spacer_32... at 99.32%

15 295bp, >spacer_33... at 99.32%

16 296bp, >spacer_36... at 98.99%

17 295bp, >spacer_37... at 98.64%

18 296bp, >spacer_39... at 99.32%

19 296bp, >spacer_40... at 99.32%

20 290bp, >spacer_42... at 98.62%

21 295bp, >spacer_43... at 98.98%

22 295bp, >spacer_44... at 98.98%

23 293bp, >spacer_46... at 98.63%

24 297bp, >spacer_48... at 98.99%

25 295bp, >spacer_50... at 98.64%

26 295bp, >spacer_51... at 99.32%

27 306bp, >spacer_53... *

28 296bp, >spacer_54... at 98.65%

29 295bp, >spacer_55... at 98.98%

30 294bp, >spacer_56... at 98.64%

31 295bp, >spacer_57... at 99.32%

32 295bp, >spacer_61... at 99.32%

33 296bp, >spacer_62... at 98.65%

34 295bp, >spacer_64... at 98.64%

35 295bp, >spacer_65... at 99.32%

36 295bp, >spacer_67... at 98.98%

37 295bp, >spacer_70... at 98.98%

38 294bp, >spacer_71... at 98.98%

39 295bp, >spacer_72... at 99.32%

40 295bp, >spacer_74... at 99.32%

41 296bp, >spacer_75... at 98.99%

42 295bp, >spacer_76... at 99.32%

43 295bp, >spacer_77... at 98.64%

44 292bp, >spacer_78... at 98.63%

45 294bp, >spacer_80... at 98.30%

46 296bp, >spacer_81... at 98.99%

47 295bp, >spacer_83... at 98.98%

48 295bp, >spacer_84... at 99.32%

49 295bp, >spacer_85... at 98.98%

50 293bp, >spacer_86... at 98.63%

51 296bp, >spacer_87... at 98.99%

52 294bp, >spacer_92... at 99.32%

53 297bp, >spacer_93... at 99.66%

54 295bp, >spacer_94... at 99.32%

55 295bp, >spacer_95... at 99.32%

56 296bp, >spacer_97... at 98.99%

57 295bp, >spacer_100... at 99.32%

58 297bp, >spacer_102... at 98.99%

59 295bp, >spacer_103... at 99.32%

60 294bp, >spacer_106... at 98.98%

61 294bp, >spacer_107... at 99.32%

62 301bp, >spacer_109... at 98.67%

63 295bp, >spacer_111... at 99.32%

64 295bp, >spacer_112... at 99.32%

65 295bp, >spacer_113... at 98.98%

66 295bp, >spacer_115... at 99.32%

67 297bp, >spacer_116... at 99.66%

68 295bp, >spacer_118... at 98.98%

69 294bp, >spacer_119... at 99.32%

70 293bp, >spacer_121... at 98.63%

71 297bp, >spacer_122... at 98.32%

72 295bp, >spacer_123... at 99.32%

73 296bp, >spacer_124... at 99.32%

74 295bp, >spacer_127... at 99.32%

75 294bp, >spacer_129... at 99.32%

76 295bp, >spacer_130... at 98.98%

77 294bp, >spacer_131... at 98.98%

78 296bp, >spacer_132... at 99.66%

79 296bp, >spacer_134... at 98.99%

80 293bp, >spacer_138... at 98.29%

81 293bp, >spacer_139... at 98.63%

82 296bp, >spacer_140... at 100.00%

83 294bp, >spacer_141... at 98.30%

84 299bp, >spacer_142... at 99.00%

85 295bp, >spacer_143... at 99.32%

86 290bp, >spacer_144... at 98.62%

87 294bp, >spacer_145... at 99.66%

>Cluster 30

0 293bp, >spacer_7... at 98.29%

1 294bp, >spacer_10... at 98.98%

2 295bp, >spacer_17... at 98.98%

3 302bp, >spacer_18... *

4 295bp, >spacer_41... at 98.98%

5 302bp, >spacer_52... at 100.00%

6 297bp, >spacer_117... at 98.65%

7 294bp, >spacer_128... at 98.98%

8 295bp, >spacer_136... at 98.64%

9 294bp, >spacer_137... at 98.64%

>Cluster 31

0 296bp, >spacer_66... at 98.65%

1 298bp, >spacer_99... at 99.33%

2 302bp, >spacer_114... *

>Cluster 32

0 298bp, >spacer_88... *

>Cluster 33

0 298bp, >spacer_89... *

>Cluster 34

0 293bp, >spacer_14... at 98.29%

1 295bp, >spacer_45... at 98.31%

2 298bp, >spacer_101... *

3 295bp, >spacer_120... at 98.31%

>Cluster 35

0 297bp, >spacer_69... *

>Cluster 36

0 296bp, >spacer_49... *

>Cluster 37

0 294bp, >spacer_125... *

>Cluster 38

0 209bp, >spacer_104... *

>Cluster 39

0 208bp, >spacer_126... *

>Cluster 40

0 207bp, >spacer_135... *
