# Supplementary material for: Fine organization of genomic regions tagged to the 5S rDNA locus of the bread wheat 5B chromosome
Source: BMC Plant Biol. 2017 Nov 14;17(Suppl 1):183. doi: 10.1186/s12870-017-1120-5 (PMC5688495; doi:10.1186/s12870-017-1120-5)
Supplement: Supplementary file 6 — The MUSCLE Alignment of representative 5S rDNA spacers from each cluster of pool_89 and LongS1 unit type [10]. The number of spacer sequences in cluster indicated in brackets. The conserved sites indicated as asterisk. The position of Long89_F and Long89_R primers used to obtain the FISH probe indicated by grey colour. (DOCX 13 kb) [file 12870_2017_1120_MOESM6_ESM.docx]

**Additional File 6. The MUSCLE Alignment of representative 5S rDNA spacers from each cluster of pool_89 and LongS1 unit type** [10]. The number of spacer sequences in cluster indicated in brackets. The conserved sites indicated as asterisk. The position of Long89_F and Long89_R primers used to obtain the FISH probe indicated by grey colour.

89_cluster_0_(15) TTTTATAATTA-TTTTTTGCGCCTTGTGACAAACATGTCGCACGTGCGCGATATATATTA

89_cluster_1_(10) TTTTATAATTACTTTTTTGCGCCTTGTGACAAACATGTCGCACGTGCGCGATATATATTA

LongS1_AF150605.1_T.aestivum TTTTATAATTA-TTTTTTGCGCCTTGTGACAAACATGTCGCACGTGCGCGATATATATTA

89_cluster_2_(2) TTTTATAA-TA-TTTTTTGCGCCTTGTGACAAACATGTCGCACGTGCGCGATATATATTA

******** ** ************************************************

89_cluster_0_(15) ATCCCGTTATATTATGTTTGACGTTTGCGATATGTTTAAGCTCGATGCTCATTGCTCGCG

89_cluster_1_(10) ATCCCGTTATATTATGTTTGACGTTTGCGATATGTTTAAGCTCGATGCTCATTGCTCGCG

LongS1_AF150605.1_T.aestivum ATCCCGTTATATTATGTTTGACGTTTGCGATATGTTTAAGCTNGATGCTCATTGCTCGCG

89_cluster_2_(2) ATCCCGTTATATTATGTTTGACGTTTGCGATATGTTTAAGCTCGATGCTCATTGCTCGCG

****************************************** *****************

Long89_F primer (5’-3’)

89_cluster_0_(15) CGTCTTGGGGCGGCTTTGTGGC-GCGA-AGAGCGCGTTCTGAAAAGGGGTGG-AAAAAAC

89_cluster_1_(10) CGTCTTGGGGCGGCTTGTTGGCGGCGAGAAAGCGCGTTCTG-AAAGGGGTGG-AAAAAAC

LongS1_AF150605.1_T.aestivum CGTCTTGGGGCGGCTTTGTGGC-GCGA-AGAGCGCGTTCTG-AAAGGGGTGG-AAAAAAC

89_cluster_2_(2) CGTCTTGGGGCGGCTTTGTGGC-GCGA-AGAGCGCGTTCTG-AAAGGGGTGGAAAAAAAC

**************** **** **** * *********** ********** *******

89_cluster_0_(15) TCGTGTTGCTGC-GGTATGGAGGGAGGGGTGGAAACCGTGGAAAACTCGTCTCCGTGATT

89_cluster_1_(10) TCGTGTTGCTGC-GGTATGGAGGGAGGGGTGGAAACCGTGGAAAACTCGTCTCCGTGATT

LongS1_AF150605.1_T.aestivum TCGTGTTGCTGC-GGTATGGAGGGAGGGGTGGAAACCGTGGAAAACTCGTCTCCGTGATT

89_cluster_2_(2) TCGTGTTGCTGCGGGTATGGAGGGAGGGGTGGAAACCGTGGAAAACTCGTCTCCATGATT

************ ***************************************** *****

Long89_R primer (3’-5’)

89_cluster_0_(15) GAGCGGGAGAGTAAGTAGTATAGGACATTATCCATTGTTAGGGAACGGTTGTAATGGTAG

89_cluster_1_(10) GAGCGGGAGAGTAAGTAGTATAGGACATTATCCATTGTTAGGGAACGGTTGTAATGGTAG

LongS1_AF150605.1_T.aestivum GAGCGGGAGAGTAAGTAGTATAGGACATTATCCATTGTTAGGGAACGGTTGTAATGGTAG

89_cluster_2_(2) GAGCGGGAGAGTAAGTAGTATAGGACATTATCCATTGTTAGGGAACGGTTGTAATGGTAG

************************************************************

89_cluster_0_(15) TGAGAATGTAGAATCGTTGCTTGGAGC-GACCTGGGAGTGGCAAGCAATAAGGGACGAAG

89_cluster_1_(10) TGAGAATGTAGAATCGTC-TTTGGAGCGGACCTGGGAGTGGCAAGC-ATAAGGGACGAAG

LongS1_AF150605.1_T.aestivum TGAGAATGTAGAATCGTC-TTTGGAGCGGACCTGGGAGTGGCAAGC-ATAAGGGACGAAG

89_cluster_2_(2) TGAGAATGTAGAATCGTC-TTTGGAGCGGACCTGGGAGTGGCAAGC-ATAAGGGACGAAG

***************** ******* ****************** *************

89_cluster_0_(15) ACGGGGAAACATGTGC

89_cluster_1_(10) ACGGGGAAACATGT-C

LongS1_AF150605.1_T.aestivum ACGGGGAAACATGT-C

89_cluster_2_(2) ACGGGGAAACATGT-C

************** *
