# Supplementary material for: Fine organization of genomic regions tagged to the 5S rDNA locus of the bread wheat 5B chromosome
Source: BMC Plant Biol. 2017 Nov 14;17(Suppl 1):183. doi: 10.1186/s12870-017-1120-5 (PMC5688495; doi:10.1186/s12870-017-1120-5)
Supplement: Supplementary file 7 — The MUSCLE Alignment of representative 5S rDNA spacers from each tree brunch of pool_52 with ShortA1, ShortA2 and ShortG1 types of 5S rDNA units [10]. The number of spacer sequences in cluster indicated in brackets. The conserved sites indicated as asterisk. The position of Short52_F and Short52_R primers used to obtain the FISH probe indicated by grey colour. (DOCX 13 kb) [file 12870_2017_1120_MOESM7_ESM.docx]

**Additional File 7. The MUSCLE Alignment of representative 5S rDNA spacers from each tree brunch of pool_52 with ShortA1, ShortA2 and ShortG1 types of 5S rDNA units [10]**. The number of spacer sequences in cluster indicated in brackets. The conserved sites indicated as asterisk. The position of Short52_F and Short52_R primers used to obtain the FISH probe indicated by grey colour.

52_cluster_29_(88) TTTT--TAATTTTTTTTCGCGCCGCTTGCAAAACAAAACGCACGTGTAAGTAATATATTT

ShortA2_AF150596.1_T.aestivum TTTT--TAATTTTTTTC-GCGCCGCTTGCAAAACAAAACGCACGTGTAAGTAATATATTT

52_cluster_9_(2) TTTT---ATTTATTTTTTGCACGACGTGCAAAACAAAACGCACGTGTGCGGCATATATTT

ShortA1_AF150511.1_T.monococcum TTTT--TAATTATTCTT-GCGTGACGTGCAAAACAAAACGCACGTGCGCGGCATATATTT

52_cluster_39_(1) TTTTAAAAATTTTTTTT-GCGCGGTGTGCAAAAGAAAACTCACGGCCGCGACATATATTT

ShortG1_AF150603.1_T.aestivum TTTT--TAATTTTTTTT-GCGCGGCGTGCAAAAGAAAACTCACGGCCACGACATATATTT

**** * ** ** * ** ******* ***** **** * ********

52_cluster_29_(88) ACCGTGTTTTATTATTTTGCACGAGTGCGGTAAGTCATAGCTGGGTGCTCACGATTCACG

ShortA2_AF150596.1_T.aestivum ACCGTGTTTTATTATTTTGCACGAGTGCGGTAAGTCATAGCTGGGTGCTCACGATTCACG

52_cluster_9_(2) ACCACGTTTTATTATTTTGCATGTTCGCGGTAAGTTTTAGCTTGTTGCTCATTATTCACG

ShortA1_AF150511.1_T.monococcum ACCACGTTTCATTATTTTGCACGTTTGCGGTAAGTTTTAGCTCGTTGCTCATGATTCACG

52_cluster_39_(1) ACCACGTTTTA-TATTTTGCACGTTTGCGGTAAGTTTTAGCTCGTTGGTCATTATTCACG

ShortG1_AF150603.1_T.aestivum ACCACGTTTTATTATTTTGCACGTTTGCGGTAAGTTTTAGCTCGTTGGTCATTATTCGCG

*** **** * ********* * ********* ***** * ** *** **** **

Short52_F Primer (5’-3’)

52_cluster_29_(88) GGTCCAGCGTCGGCGTTGTGGCGCGGCAAGCGT---------------------------

ShortA2_AF150596.1_T.aestivum GGTCCAGCGTCGGCGTTGTGGCGCGGCAAGCGT---------------------------

52_cluster_9_(2) CGTCTAGCGGCGGCGTTGTGGCGCGGGAAGCGCGTTCGGGAAGGGGTCGGAACCTTGGTA

ShortA1_AF150511.1_T.monococcum CGTGTAGCG---------------------------------------------------

52_cluster_39_(1) CGTGTAGCG---------------------------------------------------

ShortG1_AF150603.1_T.aestivum CGTGTAGCG---------------------------------------------------

** ****

52_cluster_29_(88) -----GCACTGGTGCGGTTGAGAGGGAGGGGTGGAAACCGCGTTAAACTCGTCTCCGTAG

ShortA2_AF150596.1_T.aestivum -----GCACTGGTGCGGTTGAGAGGGAGGGGTGGAAACCGCGTTAAACTCGTCTCCGTAG

52_cluster_9_(2) AATAGGCGCTGGTGCGGTTGAGATGGAGGGGTGGAAACCGTGGTAAACTTGTCTCCGTGG

ShortA1_AF150511.1_T.monococcum ------------------------------------------------------------

52_cluster_39_(1) ------------------------------------------------------------

ShortG1_AF150603.1_T.aestivum ------------------------------------------------------------

Short52_R Primer (3’-5’)

52_cluster_29_(88) TTGAGAGGGAGCGGCCAAAGCAATGTGCAATC-GTCTTTGTAGTGGAGCTGGGAGGGGCA

ShortA2_AF150596.1_T.aestivum TTGAGAGGGAGCGGCCAAAGCAATGTACAATC-GTCTTTGTAGTGGAGCTGGGAGGGGCA

52_cluster_9_(2) TTGAGCGGCAGCGGCGAAAGCAAAGTACAATCTTTTTTTGTAGTGGAGCTGGGAGGGGCA

ShortA1_AF150511.1_T.monococcum ----------GCGGGCAAAGCAAAGTGCAACC-GTGTGTGTAGTGGAGCTGGGAGGGGCA

52_cluster_39_(1) ----------GCGGGCAAAGCAAAGTACAATC-GTCTTTGTAGTGGAGCTGGGAGGGGCA

ShortG1_AF150603.1_T.aestivum ----------GCGGGCAAAGCAAAGTACAATC-GTCTTTGCAGTGGAGCTGGGAGGGGCA

**** ******* ** *** * * * ** *******************

52_cluster_29_(88) AGGATAAGGGACGAAGACCGGGGGTAACATGTC

ShortA2_AF150596.1_T.aestivum AGGATAAGGGACGAAGA-CCGGGATAACATGTC

52_cluster_9_(2) AGCATAAGGGACGAAGA-TGGGGCTAACATGTC

ShortA1_AF150511.1_T.monococcum AGCATAAGGGACGAAGA-CGGGGGTAACATGTC

52_cluster_39_(1) AGCATAAGGGACGAAGA-CGGGGGTAACATGTC

ShortG1_AF150603.1_T.aestivum AGCATAAGGGACGAAGA-CGGGGGTAACATGTC

** ************** *** *********
